# ﻿Hidden diversity of rock geckos within the *Cnemaspissiamensis* species group (Gekkonidae, Squamata): genetic and morphological data from southern Thailand reveal two new insular species and verify the phylogenetic affinities of *C.chanardi* and *C.kamolnorranathi*

**DOI:** 10.3897/zookeys.1125.94060

**Published:** 2022-10-21

**Authors:** Natee Ampai, Attapol Rujirawan, Siriporn Yodthong, Korkhwan Termprayoon, Bryan L. Stuart, Perry L. Wood Jr, Anchalee Aowphol

**Affiliations:** 1 Department of Biology, Faculty of Science, Srinakharinwirot University, Bangkok, 10110 Thailand Srinakharinwirot University Bangkok Thailand; 2 Department of Zoology, Faculty of Science, Kasetsart University, Bangkok, 10900 Thailand Kasetsart University Bangkok Thailand; 3 Department of Biology, Faculty of Science, Thaksin University, Pa Phayom, Phattalung, 93210 Thailand Thaksin University Phattalung Thailand; 4 Section of Research & Collections, North Carolina Museum of Natural Sciences, Raleigh, NC, USA Section of Research & Collections, North Carolina Museum of Natural Sciences Raleigh United States of America; 5 Department of Ecology and Evolutionary Biology, University of Michigan, Ann Arbor, MI, USA University of Michigan Ann Arbor United States of America

**Keywords:** *Cnemaspis*, integrative taxonomy, Island, phylogeny, Thailand

## Abstract

Two new insular rock geckos in the genus *Cnemaspis* are described from Ko Samui in Surat Thani Province and Ko Similan in Phang-nga Province, southern Thailand, based on a combination of morphological and mitochondrial NADH dehydrogenase subunit 2 (ND2) data. Both new species represent divergent lineages within the *Cnemaspissiamensis* species group. *Cnemaspissamui***sp. nov.** is distinguished from other species in the group by having eight or nine supralabial and infralabial scales; 5–8 pore-bearing precloacal scales in males, pores rounded; 25–27 paravertebral tubercles, arranged randomly; 22–25 subdigital lamellae under 4^th^ toe; enlarged median subcaudal scale row present; gular region, abdomen, limbs and subcaudal region yellowish only in males, and uncorrected pairwise divergences of 8.86–26.83% from all other species in the *C.siamensis* species group. *Cnemaspissimilan***sp. nov.** is distinguished from other species in the group by having eight or nine supralabial and seven or eight infralabial scales; one pore-bearing precloacal scale in males, pore rounded; 24 or 25 paravertebral tubercles, arranged randomly; 23 or 24 subdigital lamellae under 4^th^ toe; no enlarged median subcaudal scale row; pale yellow reticulum on head, neck, flanks, belly and limbs in male only, and uncorrected pairwise divergences of 9.34–27.11% from all other species in the *C.siamensis* species group. *Cnemaspissamui***sp. nov.** is found along granitic rocky stream outcrops of Hin Lad Waterfall, Ko Samui, Gulf of Thailand, while *Cnemaspissimilan***sp. nov.** occurs in granitic rocky outcrops near Ao Nguang Chang Bay, Ko Similan, Andaman Sea. The phylogenetic analyses confirmed that *C.chanardi* and *C.kamolnorranathi* are also nested within the *C.siamensis* species group, as previously hypothesized from morphology and color pattern characters.

## ﻿Introduction

The rock gecko genus *Cnemaspis* Strauch, 1887 is one of the most diverse reptile genera, with 192 recognized species known to date (Uetz et al. 2022). The genus has a geographically widespread distribution, ranging from South Asia to Southeast Asia, and is composed of two separate clades based on multi-locus phylogenetic analyses (Gamble et al. 2012, 2015; Pyron et al. 2013; Karunarathna et al. 2019; Malonza and Bauer 2022). The 64 currently described Southeast Asian species of *Cnemaspis* represent a monophyletic group, include many species with specializations for various rocky habitats (Grismer et al. 2010, 2014; Nguyen et al. 2020), and are distributed in Myanmar, Thailand, Laos, Cambodia, Vietnam, Malaysia and Indonesia (Bauer and Das 1998; Das 2005; Grismer and Ngo 2007; Grismer et al. 2010, 2014, 2020; Kurita et al. 2017; Riyanto et al. 2017; Wood et al. 2017; Ampai et al. 2019, 2020; Lee et al. 2019; Nashriq et al. 2022). In Thailand, 19 named species of Southeast Asian *Cnemaspis* (Grismer et al. 2010, 2014, 2020; Wood et al. 2017; Ampai et al. 2019, 2020; Uetz et al. 2022) occur throughout much of the country’s mainland and adjacent offshore islands (Grismer et al. 2014, 2020; Wood et al. 2017; Ampai et al. 2019, 2020; Lee et al. 2019).

Historically, the taxonomy and systematics of Thai *Cnemaspis* depended solely on data from morphology and color pattern characteristics (e.g., Smith 1925; Taylor 1963; Bauer and Das 1998; Grismer et al. 2010). During the past decade, integrative taxonomic approaches that included morphological characteristics, ecological data, and molecular genetics (e.g., Grismer et al. 2014, 2020; Wood et al. 2017; Ampai et al. 2019, 2020; Lee et al. 2019) have been used to address and resolve previous taxonomic issues (Wood et al. 2017). Thai *Cnemaspis* species are assigned to four species groups based on morphological character state differences and genetics (Grismer et al. 2014; Ampai et al. 2019, 2020), these being the *affinis* group, the *chanthaburiensis* group, the *kumpoli* group, and the *siamensis* group. Of these, the *siamensis* group shows the highest species richness with 12 recognized species in Thailand, including *C.adangrawi*Ampai et al. 2019, *C.chanardi*Grismer et al. 2010, *C.huaseesom*Grismer et al. 2010, *C.kamolnorranathi*Grismer et al. 2010, *C.lineatubercularis*Ampai et al. 2020, *C.omari*Grismer et al. 2014, *C.phangngaensis*Wood et al. 2017; *C.punctatonuchalis*Grismer et al. 2010, *C.selenolagus*Grismer et al. 2020, *C.siamensis* (Smith, 1925), *C.thachanaensis*Wood et al. 2017, and *C.vandeventeri*Grismer et al. 2010. Only one member of the *siamensis* group, *C.roticanai* Grismer & Chan, 2010, occurs outside of Thailand, where it occurs on Langkawi Island, Malaysia. Within the *siamensis* group, *C.chanardi* and *C.kamolnorranathi* remain the least known species, in part due to a lack of genetic data from their type localities.

We conducted field surveys for *Cnemaspis* during 2015–2020 at five localities in southern Thailand. Morphological and mitochondrial DNA data analyses revealed that the *Cnemaspis* samples from Ko (= island) Samui in the Gulf of Thailand and Ko Similan in the Andaman Sea differed from all known congeners of Thai *Cnemaspis*. In addition, we obtained samples from the type localities of *C.chanardi* and *C.kamolnorranathi*. Herein, the two distinct insular populations of *Cnemaspis* are described as new species and genetic data are used to verify the phylogenetic placements of *C.chanardi* and *C.kamolnorranathi* within the *C.siamensis* group.

## ﻿Materials and methods

### ﻿Taxon sampling and specimen collection

Fieldwork was conducted at five different localities in southern Thailand including (1) Pathio District, Chumphon Province in June 2017, (2) Kanchanadit District, Surat Thani Province in January 2019, (3) Nayong District, Trang Province in May 2016, December 2017, and July 2019, (4) Ko Samui, Surat Thani Province in September 2015, July 2018, and August 2020, and (5) Ko Similan, Mu Ko Similan National Park, Phang-nga Province in March 2018. Sampling was conducted by using visual encounter surveys both during the day (1000–1800 h) and at night (1900–2200 h). Ecological data (air temperature and relative humidity) were collected using a Kestrel 4000 Weather Meter. Habitat preferences (e.g., microhabitat, substrate type and habitat use) were also recorded. Geographical coordinates and elevation were taken using a Garmin GPSMAP 64s. At each locality, specimens were photographed and euthanized by cardiac injection of tricaine methane sulfonate (MS-222) solution (Simmons 2015). Liver samples were removed from euthanized specimens for molecular analysis, preserved in 95% ethanol, and stored at -20 °C. Voucher specimens were then fixed in 10% formalin and later transferred to 70% ethanol for long-term storage. Specimens and tissue samples were deposited in the herpetological collection at the Zoological Museum of Kasetsart University, Bangkok, Thailand (**ZMKU**) . All specimens of the *C.siamensis* group, including the type specimens of *C.adangrawi*, *C.chanardi*, *C.huaseesom*, *C.lineatubercularis*, *C.niyomwanae*, *C.punctatonuchalis*, and *C.vandeventeri*, were examined as comparative material (Appendix I) in the holdings of ZMKU and the Thailand Natural History Museum, Pathum Thani, Thailand (**THNHM**). Additional data were obtained from the original species descriptions of Thai *Cnemaspis* (Smith, 1925; Grismer et al. 2010, 2014, 2020; Wood et al. 2017; Ampai et al. 2019, 2020).

### ﻿Molecular genetic study and phylogenetic analyses

Genomic DNA from 21 individuals of *Cnemaspis* (*C.adangrawi*, *N* = 2; *C.chanardi*, *N* = 4; *C.kamolnorranathi*, *N* = 5; *C.siamensis*, *N* = 2; Ko Samui samples, *N* = 4; and Ko Similan samples, *N* = 4) was extracted from liver tissue (Table 1) using the Qiagen DNAeasy^TM^ Blood & Tissue Kit (Valencia, CA, USA). A fragment of mitochondrial (mt) DNA encoding the NADH dehydrogenase subunit 2 (ND2) gene and parts of its flanking tRNAs Trp, Ala, Asn and Cys was amplified by polymerase chain reaction (PCR) with an initial denaturation (95 °C, 2 min) followed by 33 cycles of denaturation (95 °C, 35s), annealing (52 °C, 35s), and extension (72 °C, 35s) and the light strand primer MetF1(L4437b; 5’-AAGCAGTTGGGCCCATACC-3’; Macey et al. 1997) and heavy strand primer CO1R1 (H5934; 5’-AGRGTGCCAATGTCTTTGTGRTT-3’; Macey et al. 1997). PCR products were purified by the NucleoSpin Gel and PCR Clean-up Kit (Machery-Nagel Inc.) and sequenced using the amplifying primers on an ABI 3730XL automatic sequencer (Sango Biotech Inc, Shanghai, China). The generated DNA sequences were edited and aligned using Geneious R11 (Biomatters, Ltd, Auckland, New Zealand). The newly generated sequences were deposited in GenBank under accession numbers ON843665–ON843685 (Table 1).

**Table 1.** Voucher information, including locality, collection numbers, GenBank accession numbers and reference for the specimens used in the phylogenetic analyses. Voucher abbreviations are as follows: Monte L. Bean Life Science Museum at Brigham Young University (**BYU**), California Academy of Sciences (**CAS**), the Field Museum of Natural History, Chicago, Illinois, USA (**FMNH**), La Sierra University Herpetological Collection (**LSUHC**), the Thailand Natural History Museum, Pathum Thani, Thailand (**THNHM**), Universiti Sains Malaysia Herpetological Collection at the Universiti Sains Malaysia, Penang, Malaysia (**USMHC**), and Zoological Museum of Kasetsart University (**ZMKU**).

Species	Locality	Collection number	GenBank accession number	Reference
**Outgroup**				
*Cyrtodactylusbokorensis*	Cambodia, Kampot	FMNH 263228	KT013107	Grismer et al. 2015b
*Dixoniusmelanostictus*	Thailand (captive)	No number	HM997153	Ziegler et al. 2016
*Dixoniussiamensis*	Cambodia, Pursat Province, Phnom Aural	LSUHC 7328	EU054299	Ziegler et al. 2016
*Gekkogecko*	Myanmar, Ayeyarwady Division, Myaungmya District	CAS 204952	JN019052	Rösler et al. 2011
*Gehyramutilata*	Cambodia, Pursat Province, Phnom Aural	LSUHC 7379	JN393914	Wood et al. 2017
*Hemidactylusfrenatus*	Myanmar, Tanintharyi Division, Kaw Thaung District	CAS 229633	HM559629	Bauer et al. 2010
*Hemidactylusgarnotii*	Myanmar, Mon State, Kyait Hti Yo Wildlife Sanctuary	CAS 222276	EU268364	Bauer et al. 2010
**Ca Mau Clade**				
*Cnemaspisboulengerii*	Vietnam, Ca Mau Province, Con Dao Archipelago	LSUHC 9278	KM024710	Grismer et al. 2014
		LSUHC 9279	KM024711	
*Cnemaspispsychedelica*	Vietnam, Ca Mau Province, Hon Khoai Island	LSUHC 9243	KM024827	Wood et al. 2017
		LSUHC 9244	KM024828	
***chanthaburiensis* group**				
*Cnemaspisaurantiacopes*	Vietnam, Kien Giang Province, Hon Dat Hill	LSUHC 8610	KM024692	Grismer et al. 2014
		LSUHC 8611	KM024693	
*Cnemaspiscaudanivea*	Vietnam, Kien Giang Province, Hon Tre Island	LSUHC 8582	KM024714	Grismer et al. 2014
*Cnemaspischanthaburiensis*	Cambodia, Pursat Province, Phnom Dalai	LSUHC 9338	KM024716	Grismer et al. 2014
*Cnemaspislineogularis*	Thailand, Prachuap Khiri Khan Province, Kui Buri District, Wat Khao Daeng	BYU 62535	KY091231	Wood et al. 2017
		ZMKU R 00728	KY091233	
*Cnemaspisneangthyi*	Cambodia, Pursat Province, O’Lakmeas	LSUHC 8515	KM024767	Grismer et al. 2014
		LSUHC 8516	KM024768	
*Cnemaspisnuicamensis*	Vietnam, An Giang Province, Nui Cam Hill	LSUHC 8646	KM024775	Grismer et al. 2014
		LSUHC 8647	KM024776	
		LSUHC 8648	KM024777	
***kumpoli* group**				
*Cnemaspisbiocellata*	Malaysia, Perlis, Kuala Perlis	LSUHC 8789	KM024707	Grismer et al. 2014
		LSUHC 8817	KM024708	
	Malaysia, Perlis, Gua Kelam	LSUHC 8818	KM024709	
*Cnemaspiskumpoli*	Malaysia, Perlis, Perlis State Park	LSUHC 8847	KM024745	Grismer et al. 2014
		LSUHC 8848	KM024746	
*Cnemaspismonachorum*	Malaysia, Kedah, Langkawi Archipelago, Pulau Langkawi	LSUHC 9114	KM024754	Grismer et al. 2014
		LSUHC 10807	KM024755	
*Cnemaspisniyomwanae*	Thailand, Trang Province, Thum Khao Ting	LSUHC 9568	KM024773	Grismer et al. 2014
		LSUHC 9571	KM024774	
*Cnemaspistarutaoensis*	Thailand, Satun Province, Mueang Satun District, Ko Tarutao	ZMKU R 00761	MK862117	Ampai et al. 2019
		ZMKU R 00763	MK862118	
		ZMKU R 00764	MK862119	
***argus* group**				
*Cnemaspisargus*	Malaysia, Terengganu, Gunung Lawit	LSUHC 8304	KM024687	Grismer et al. 2014
		LSUHC 10834	KM024688	
*Cnemaspiskarsticola*	Malaysia, Kelantan, Gunung Reng	LSUHC 9054	KM024736	Grismer et al. 2014
		LSUHC 9055	KM024737	
***affinis* group**				
*Cnemaspisaffinis*	Malaysia, Penang, Pulau Pinang	LSUHC 6787	KM024682	Grismer et al. 2014
*Cnemaspisgrismeri*	Malaysia, Perak, Lenggong	LSUHC 9969	KM024722	Grismer et al. 2014
*Cnemaspishangus*	Malaysia, Pahang, Bukit Hangus	LSUHC 9358b	KM024728	Grismer et al. 2014
*Cnemaspisharimau*	Malaysia, Kedah, Gunung Jeri	LSUHC 9665	KM024730	Grismer et al. 2014
*Cnemaspismahsuriae*	Malaysia, Kedah, Pulau Langkawi, Gunung Raya	LSUHC 11829	KT250634	Grismer et al. 2015a
*Cnemaspismcguirei*	Malaysia, Perak, Bukit Larut	LSUHC 8853	KM024751	Grismer et al. 2014
*Cnemaspisnarathiwatensis*	Malaysia, Perak, Belum-Temengor, Sungai Enam	USMHC 1347	KM024762	Grismer et al. 2014
		USMHC 1348	KM024763	
***siamensis* group**				
*Cnemaspisadangrawi*	Thailand, Satun Province, Mueang Satun District, Ko Adang	ZMKU R 00767	MK862112	Ampai et al. 2019
		ZMKU R 00768	ON843665	This study
		THNHM 28207	MK862113	Ampai et al. 2019
		ZMKU R 00770	MK862114	
	Thailand, Satun Province, Mueang Satun District, Ko Rawi	ZMKU R 00774	ON843666	This study
		ZMKU R 00775	MK862115	Ampai et al. 2019
		ZMKU R 00776	MK862116	
*Cnemaspischanardi*	Thailand, Trang Province, Nayong District	ZMKU R 00988	ON843675	This study
		ZMKU R 00989	ON843676	
		ZMKU R 00990	ON843677	
		ZMKU R 00991	ON843678	
*Cnemaspishuaseesom*	Thailand, Kanchanaburi Province, Sai Yok National Park	LSUHC 9455	KM024733	Grismer et al. 2014
		LSUHC 9457	KM024734	
		LSUHC 9458	KM024735	
*Cnemaspiskamolnorranathi*	Thailand, Surat Thani Province, Kanchanadit District, Tai Rom Yen National Park	ZMKU R 00992	ON843679	This study
		ZMKU R 00993	ON843680	
		ZMKU R 00994	ON843681	
		ZMKU R 00995	ON843682	
		ZMKU R 00996	ON843683	
*Cnemaspislineatubercularis*	Thailand, Nakhon Si Thammarat Province, Lan Saka District, Wang Mai Pak Waterfall	ZMKU R 00825	MT112890	Ampai et al. 2020
		ZMKU R 00828	MT112891	
		ZMKU R 00829	MT112892	
		ZMKU R 00830	MT112893	
*Cnemaspisomari*	Thailand, Satun Province, Phuphaphet Cave	LSUHC 9565	KM024780	Grismer et al. 2014
	Malaysia, Perlis, Perlis State Park	LSUHC 9978	KM024779	
*Cnemaspisphangngaensis*	Thailand, Phang-nga Province, Mueang Phang-nga District, Khao Chang, Phung Chang Cave	BYU 62537	KY091234	Wood et al. 2017
		BYU 62538	KY091235	
*Cnemaspispunctatonuchalis*	Thailand, Prachaup Khiri Khan Province, Thap Sakae	BYU 62539	KY091236	Wood et al. 2017
		BYU 62540	KY091237	
*Cnemaspisroticanai*	Malaysia, Kedah, Pulau Langkawi, Gunung Raya	LSUHC 9430	KM024829	Grismer et al. 2014
		LSUHC 9431	KM024830	
		LSUHC 9439	KM024831	
*Cnemaspissamui* sp. nov.	Thailand, Surat Thani Province, Ko Samui District, Ko Samui, Hin Lad Waterfall	ZMKU R 00966	ON843667	This study
		ZMKU R 00967	ON843668	
		ZMKU R 00968	ON843669	
		ZMKU R 00974	ON843670	
*Cnemaspissimilan* sp. nov.	Thailand, Phang-nga Province, Tai Mueang District, Mu Ko Similan National Park, Ko Similan, Ao Nguang Chang	ZMKU R 00984	ON843671	This study
		ZMKU R 00985	ON843672	
		ZMKU R 00986	ON843673	
		ZMKU R 00987	ON843674	
*Cnemaspisselenolagus*	Thailand, Ratchaburi Province, Suan Phueng District, Khao Laem Mountain	ZMMU R 16391	MW051887	Grismer et al. 2020
		AUP 00767	MW051888	
*Cnemaspissiamensis*	Thailand, Chumpon Province, Pathio District	LSUHC 9474	KM024838	Grismer et al. 2014
		LSUHC 9485	KM024839	
		ZMKU R 00997	ON843684	This study
		ZMKU R 00998	ON843685	
*Cnemaspisthachanaensis*	Thailand, Surat Thani Province, Tha Chana District, Tham Khao Sonk Hill	BYU 62542	KY091239	Wood et al. 2017
		BYU 62543	KY091243	
		BYU 62544	KY091244	
*Cnemaspisvandeventeri*	Thailand, Ranong Province, Suk Saran District, Naka	BYU 62541	KY091238	Wood et al. 2017

Homologous sequences of 68 *Cnemaspis* and the seven outgroups *Cyrtodactylusbokorensis* Murdoch, Grismer, Wood, Neang, Poyarkov, Tri, Nazarov, Aowphol, Pauwels, Nguyen & Grismer, 2019, *Dixoniusmelanostictus* (Taylor, 1962), *Dixoniussiamensis* (Boulenger, 1898), *Gehyramutilata* Wiegmann, 1834, *Gekkogecko* (Linnaeus, 1758), *Hemidactylusfrenatus* Duméril & Bibron, 1836 and *Hemidactylusgarnotii* Duméril & Bibron, 1836 (following Wood et al. 2017; Ampai et al. 2020) were downloaded from GenBank. These were aligned to the 21 newly generated sequences of *Cnemaspis* using the MUSCLE plug-in as implemented in Geneious R11 (Biomatters, Ltd, Auckland, New Zealand). The aligned dataset was partitioned into four partitions consisting of ND2 codon positions and the flanking tRNAs. Molecular phylogenetic relationships were estimated using Bayesian inference (BI) and maximum likelihood (ML). The BI was implemented in MrBayes v3.2.7a (Ronquist et al. 2012) on XSEDE (CIPRES; Miller et al. 2010). The best-fit model of sequence evolution for each partition was estimated using the Bayesian Information Criterion (BIC) as implemented in PartitionFinder2 on XSEDE (Lanfear et al. 2016). The selected models were GTR+I+Γ for each ND2 codon partition and HKY+I+Γ for tRNAs. The BI analysis was performed as two simultaneous runs, each with four Markov chains (three heated and one cold chain), using the default priors and chain temperature set to 0.1 for 20,000,000 generations, with trees sampled every 2,000 generations from the Markov Chain Monte Carlo (MCMC). The first 25% of each run was discarded as burn-in using the “sumt” command. The convergence of the two simultaneous runs, stationary state of each parameter, and the effective sample sizes were evaluated by visualizing the log file in Tracer v1.6 (Rambaut et al. 2014). Nodes with Bayesian posterior probabilities support (BPP) of ≥ 0.95 were considered well-supported (Huelsenbeck and Ronquist 2001; Wilcox et al. 2002).

The ML analysis was implemented using the IQ-TREE web server (Nguyen et al. 2015; Trifinopoulos et al. 2016). The best-fit model of evolution for each partition was estimated using IQ-TREE’s ModelFinder function (Kalyaanamoorthy et al. 2017). Based on the Bayesian Information Criterion (BIC), the TIM+F+I+G4 was the best-fit model for 1^st^, 2^nd^, 3^rd^ codon partitions and HKY+F+G4 for tRNAs. The ultrafast bootstrap analysis (UFB; Minh et al. 2013; Hoang et al. 2017) using 10,000 bootstrap pseudo-replicates was used to construct a final consensus ML tree. Nodes with ultrafast bootstrap support (UFB) of ≥ 95 were considered well-supported (Minh et al. 2013). The 50% majority-rule consensus of sampled trees from the BI analysis and the most likely tree in the ML analysis were visualized and edited in FigTree v1.4.4 (Rambaut 2018). Uncorrected pairwise sequence divergences were estimated using a *p*-distance method with the pairwise deletion option in MEGA 11.0.11 (Tamura et al. 2021).

### ﻿Morphological measurement and analyses

Coloration and pattern in life was determined by examination of digital images taken of living specimens of all possible age classes prior to preservation. Morphological and meristic data were taken by the first author on the left side of preserved specimens for symmetrical characters, when possible, using digital Mitutoyo CD-6” ASX Digimatic Calipers to the nearest 0.1 mm under a Nikon SMZ745 dissecting microscope. Three body-size classes were established by snout-vent length: small size (< 35 mm), medium size (35–40 mm) and large size (> 40 mm). Only adult individuals, as determined by the presence of secondary sexual characteristics such as pore-bearing precloacal scales or hemipenes in males or visible eggs on ventral side of body or enlarged endolymphatic glands in females, were included for morphometric and meristic measurements. A total of sixteen morphological characters was scored following Grismer et al. (2020), Wood et al. (2017) and Ampai et al. (2020): snout-vent length (**SVL**, taken from tip of snout to the anterior margin of vent); tail width (**TW**, at the base of the tail immediately posterior to the postcloacal swelling); tail length (**TL**, distance from the vent to the tip of the tail, whether original or regenerated) ; forearm length (**FL**, taken on the dorsal surface from the posterior margin of the elbow while flexed 90° to the inflection of the flexed wrist) ; tibia length (**TBL**, taken on the ventral surface from the posterior surface of the knee while flexed 90° to the base of the heel); head length (**HL**, distance from the posterior margin of the retroarticular process of the lower jaw to the tip of the snout); head width (**HW**, at the angle of the jaws) ; head depth (**HD**, the maximum height of head from the occiput to the throat) ; axilla-groin length (**AG**, taken from the posterior margin of the forelimb at its insertion point on the body to the anterior margin of the hind limb at its insertion point on the body); eye diameter (**ED**, the maximum horizontal diameter of the eyeball) ; eye-ear distance (**EE**, measured from the anterior margin of the ear opening to the posterior edge of the eyeball) ; ear length (**EL**, taken from the greatest vertical distance of the ear opening) ; eye-nostril distance (**EN**, measured from the anterior most margin of the eyeball to the posterior margin of the external nares); eye-snout distance (**ES**, measured from the anterior margin of the eyeball to the tip of snout); internarial distance (**IN**, measured between the medial margins of the nares across the rostrum) and inner orbital distance (**IO**, the width of the frontal bone at the level of the anterior edges of the orbit).

Meristic characters states of scales and quantitative observations of pattern and structures were evaluated under a Nikon SMZ745 dissecting microscope. Meristic characters taken were modified from Grismer et al. (2014, 2020), Wood et al. (2017) and Ampai et al. (2020) as follows: number of supralabial (**SupL**) and infralabial (**InfL**) scales, counted from below the middle of the orbit to the rostral and mental scales, respectively; texture of scales on the anterior margin of the forearm; number of paravertebral tubercles (**PVT**) between limb insertions, counted in a straight line immediately left of the vertebral column; general size (i.e., strong, moderate, weak) and arrangement (i.e., random or linear) of dorsal body and tail tuberculation; number of subdigital lamellae beneath the fourth toe (**4TL**), counted from the base of the first phalanx to the claw; and number of postcloacal tubercles on each side of tail base. Categorical character states examined were: presence or absence of dark round spots on the nape and anterior portion of the body; the presence or absence of ocelli on the shoulder region; coloration of dorsal blotching on head, body, limbs and tail; presence or absence of a row of enlarged, widely spaced, tubercles along the ventrolateral edge of the body flank between limb insertions; number, orientation and shape of pore-bearing precloacal scales; and relative size of subcaudal and subtibial scales. Descriptions refer to right (R) and left (L) sides of the body.

Statistical analyses were used to compare differences in size and shape within the *siamensis* group, including populations from Ko Samui (*N* = 18), Ko Similan (*N* = 4) and the seven described species *C.adangrawi* (*N* = 8), *C.chanardi* (*N* = 7), *C.lineatubercularis* (*N* = 19), *C.omari* (*N* = 5), *C.phangngaensis* (*N* = 3), *C.siamensis* (*N* = 8) and *C.thachanaensis* (*N* = 6). Due to lack of available measurements, six species in the *siamensis* group (*C.huaseesom*, *C.kamolnorranathi*, *C.punctatonuchalis*, *C.roticanai*, *C.selenolagus* and *C.vandeventeri*) were not included in the morphometric analyses. All specimens were assigned to nine putative operation taxonomic units (OTUs) based on the mtDNA results: OTU1 (= Ko Samui population), OTU2 (= Ko Similan population), OTU3 (= *C.adangrawi*), OTU4 (= *C.chanardi*), OTU5 (= *C.lineatubercularis*), OTU6 (= *C.omari*), OTU7 (= *C.phangngaensis*), OTU8 (= *C.siamensis*) and OTU9 (= *C.thachanaensis*). TL (tail length) was excluded due to their different conditions (e.g., complete, broken, and regenerated). All morphological variables were adjusted for differences in ontogenetic composition by the allometric equation:

X_adj_ = log(*X*) – *b*[log(SVL) – log(SVL_mean_)]

where X_adj_ is the corrected value of the morphometric variable; *X* is the unadjusted value of dependent variable; *b* is the within-clade coefficient of the linear regression of each original character value (*X*) against SVL; SVL = snout-vent length; and SVL_mean_ = overall mean of SVL of all nine OTUs (Thorpe 1975, 1983; Turan 1999; Lleonart et al. 2000; Chan and Grismer 2021).

Univariate analyses were implemented in the Paleontological statistics software (PAST v4.07b; Hammer et al. 2001) using an analysis of variance (ANOVA) to compare morphological differentiation in traits among nine putative OTUs (OTU1–OTU9). Morphological characters with equal variances and having *p*-values less than 0.05 were subjected to a Tukey’s honestly significant difference (HSD) test to identify all pairwise comparisons among sample means for significant differences (*p* < 0.05). Moreover, multivariate analyses were performed using the platform R v3.2.1 (R Core Team 2018). A principal component analysis (PCA) using the built-in R functions: “prcomp” (R Core Team, 2018) and “ggplot2” (Wickham, 2016) was performed to find the best low-dimensional space of morphological variation in data. Principal components (PCs) with eigenvalues greater than 1.0 were retained in accordance with the criterion of Kaiser (1960). A discriminant analysis of principal components (DAPC) was applied using the “adegenet” package in R (Jombart 2008) to characterize clustering and distance in the morphospace of the two new groups compared to the seven named congeners of the *siamensis* group, as delimited by the molecular phylogenetic analyses. The DAPC relied on transformed and scaled data from the PCA as a prior step to find the linear combinations of morphological variables having the greatest between-group variance and the smallest within-group variance of linear distances (Jombart et al. 2010).

## ﻿Results

### ﻿Molecular analyses

**Figure 1.**
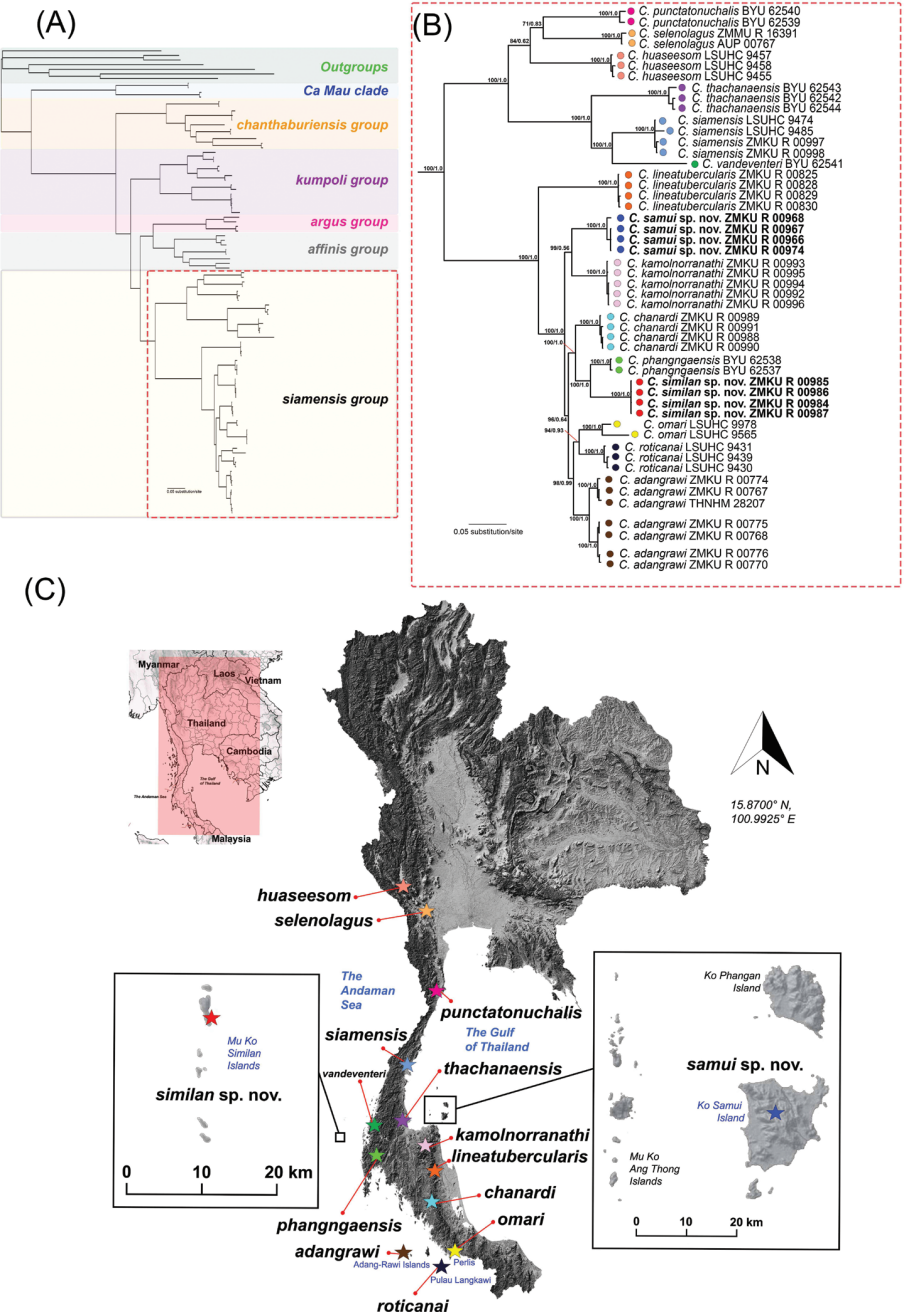
The single best tree from 10,000 Maximum likelihood bootstrap replicates based on 1,310 bp of the mitochondrial NADH dehydrogenase subunit 2 (ND2) and flanking tRNAs from geckos of the genera *Cnemaspis*, *Cyrtodactylus*, *Dixonius*, *Gekko*, *Hemidactylus* and *Gehyra***A** shown in full view **B** relevant clades of *Cnemaspissiamensis* group in close-up view **C** map illustrating the type locality of all species in the *siamensis* group. Nodal support values are ultrafast bootstrap values from maximum likelihood analysis of the same dataset followed by posterior probabilities of Bayesian analysis.

The aligned dataset contained 1,310 characters of 89 individuals of *Cnemaspis* and seven individuals of the outgroup species (Fig. 1A). Estimated base frequencies of the *Cnemaspis* dataset excluding outgroups were A = 30.52%, C = 34.65%, G = 12.59% and T = 22.24%. The BI and ML phylogenetic trees had similar topologies, with only minor differences in positions of unresolved branches (Fig. 1B). The maximum standard deviation of split frequencies among the two simultaneous BI runs was 0.016685. The average standard deviation of split frequencies among the two simultaneous BI runs was 0.002622 and ESS values were greater than or equal to 6,152 for all parameters. The maximum likelihood value of the best ML tree was lnL = –81,696.218.

*Cnemaspis* samples from Ko Samui and Ko Similan represented well-supported independent lineages (100 UFB, 1.0 BPP) and were nested within the *siamensis* group (Fig. 1B). The Ko Samui samples were well-supported for ML (99 UFB) but lacked support from BI (0.56 BPP) as the sister lineage to *C.kamolnorranathi* from its type locality at Ban Nasan District, Surat Thani Province, Thailand. The Ko Samui samples had uncorrected *p*-distances of 9.10–9.73% from *C.kamolnorranathi* and 8.86–26.83% from the other species in the *siamensis* group. The Ko Similan samples were recovered as a well-supported lineage (100 UFB, 1.0 BPP) and sister to *C.phangngaensis* (Fig. 1B). The Ko Similan samples had uncorrected *p*-distances of 8.16% from *C.phangngaensis* and 9.34–27.11% from the other species in the *siamensis* group. The Ko Samui and the Ko Similan populations had within population uncorrected *p*-distances of 0.00–1.11% and 0.00%, respectively (Table 2).

**Table 2.** Mean (min-max) uncorrected pairwise distances (%) within the *Cnemaspissiamensis* group based on 1,310 bp of the mitochondrial ND2 gene and flanking tRNAs. Number in bold are within species divergence. *N* = number of individuals.

Species	N	1	2	3	4	5	6	7	8	9	10	11	12	13	14	15
1. *Cnemaspissamui* sp. nov.	4	**0.55**														
		**(0.00–1.11)**														
2. *Cnemaspissimilan* sp. nov.	4	11.50	**0.00**													
		(11.32–11.56)	**(0.00–0.00)**													
3. *C.adangrawi*	9	9.32	11.12	**1.58**												
		(8.87–9.68)	(11.08–11.18)	**(0.00–3.01)**												
4. *C.chanardi*	4	8.92	9.40	7.80	**0.26**											
		(8.86–9.10)	(9.34–9.58)	(7.52–8.15)	**(0.00–0.32)**											
5. *C.huaseesom*	3	23.00 (22.34–23.67)	23.19 (22.72–24.10)	22.47 (22.01–23.27)	22.63 (22.10–23.58)	**0.31 (0.00–0.78)**										
6. *C.kamolnorranathi*	5	9.55 (9.10–9.73)	11.73 (11.72–11.80)	9.08 (8.86–9.44)	8.38 (8.23–8.54)	23.48 (23.13–24.10)	**0.08 (0.00–0.24)**									
7. *C.lineatubercularis*	4	14.61 (14.10–14.96)	16.15 (15.92–16.39)	14.19 (13.63–14.77)	14.04 (13.63–14.41)	24.13 (23.02–25.41)	14.55 (14.23–14.89)	**0.11 (0.00–0.25)**								
8. *C.omari*	2	10.98 (10.17–11.81)	11.42 (10.79–12.06)	8.20 (6.77–9.40)	9.36 (8.72–9.96)	24.74 (24.00–25.60)	10.81 (10.04–11.65)	16.15 (15.07–17.04)	**2.36 (0.00–4.72)**							
9. *C.phangngaensis*	2	9.68 (9.58–9.81)	8.16 (8.16–8.16)	9.00 (8.87–9.21)	7.48 (7.36–7.75)	21.69 (21.37–22.29)	10.23 (10.06–10.36)	15.62 (15.24–16.01)	10.50 (9.70–11.33)	**0.16 (0.00–0.32)**						
10. *C.punctatonuchalis*	2	23.53 (21.83–24.93)	25.24 (23.67–26.82)	23.19 (21.75–24.53)	23.74 (22.46–24.93)	16.70 (15.57–17.59)	24.34 (23.02–25.62)	23.72 (21.82–25.62)	24.79 (22.75–27.82)	24.21 (22.78–25.65)	**0.75 (0.00–1.5)**					
11. *C.roticanai*	3	10.57 (10.21–10.83)	11.21 (10.61–11.53)	7.69 (7.04–8.20)	8.83 (8.31–9.36)	24.15 (23.27–24.50)	10.23 (9.64–10.50)	14.94 (13.80–15.76)	8.34 (7.13–8.99)	9.00 (8.39–9.36)	23.14 (21.83–24.03)	**0.32 (0.00–0.69)**				
12. *C.selenolagus*	2	22.75 (22.00–23.15)	22.69 (22.48–22.91)	21.53 (21.13–21.88)	21.32 (20.97–21.72)	16.08 (15.44–17.00)	22.06 (21.84–22.28)	22.46 (21.91–22.99)	23.33 (23.02–23.57)	21.35 (21.05–21.66)	15.93 (15.29–17.13)	22.15 (21.21–22.89)	**0.28 (0.00–0.56)**			
13. *C.siamensis*	4	22.04 (20.83–23.59)	23.21 (21.73–24.88)	22.20 (21.21–23.39)	21.70 (20.64–23.11)	19.28 (18.73–19.59)	22.27 (21.12–23.83)	23.00 (22.30–24.64)	23.30 (21.21–24.58)	21.68 (20.25–23.29)	19.49 (18.12–20.70)	23.26 (21.41–24.87)	19.10 (18.72–19.55)	**0.55 (0.00–1.74)**		
14. *C.thachanaensis*	3	23.10 (22.66–23.29)	24.27 (24.12–24.50)	23.43 (22.97–23.89)	22.59 (22.35–22.89)	20.98 (20.79–21.11)	23.04 (22.91–23.19)	24.08 (23.65–24.58)	25.03 (24.88–25.24)	23.44 (23.29–23.71)	20.83 (20.05–21.57)	24.79 (24.09–25.32)	20.73 (20.49–21.00)	14.31 (13.86–15.21)	**0.67 (0.00–1.74)**	
15. *C.vandeventeri*	1	26.76 (26.56–26.83)	27.11 (27.11–27.11)	26.36 (26.15–26.42)	26.49 (26.42–26.56)	20.75 (20.66–20.80)	27.52 (27.52–27.52	25.23 (24.65–25.86)	27.36 (27.11–27.60)	26.71 (26.59–26.83)	20.89 (20.89–20.89)	27.71 (27.66–27.80)	23.24 (23.00–23.48)	13.84 (13.64–14.21)	17.45 (17.22–17.63)	**0.00 (0.00–0.00)**

*Cnemaspischanardi* and *C.kamolnorranathi* samples from their type localities (Fig. 1B, C) represented well-supported independent lineages (100 UFB, 1.0 BPP). *Cnemaspischanardi* was well-supported (100 UFB, 1.0 BPP) as sister to a clade comprised of *C.phangngaensis* and the Ko Similan population. *Cnemaspiskamolnorranathi* was recovered as the sister lineage to the Ko Samui population (99 UFB, 0.56 BPP). *Cnemaspischanardi* and *C.kamolnorranathi* had uncorrected *p*-distances of 7.36–26.56% and 8.23–27.52% from the other species in the *siamensis* group, respectively. The within population uncorrected *p*-distances of *C.chanardi* and *C.kamolnorranathi* were 0.00–0.32% and 0.00–0.24%, respectively.

### ﻿Morphological analyses

**Figure 2.**
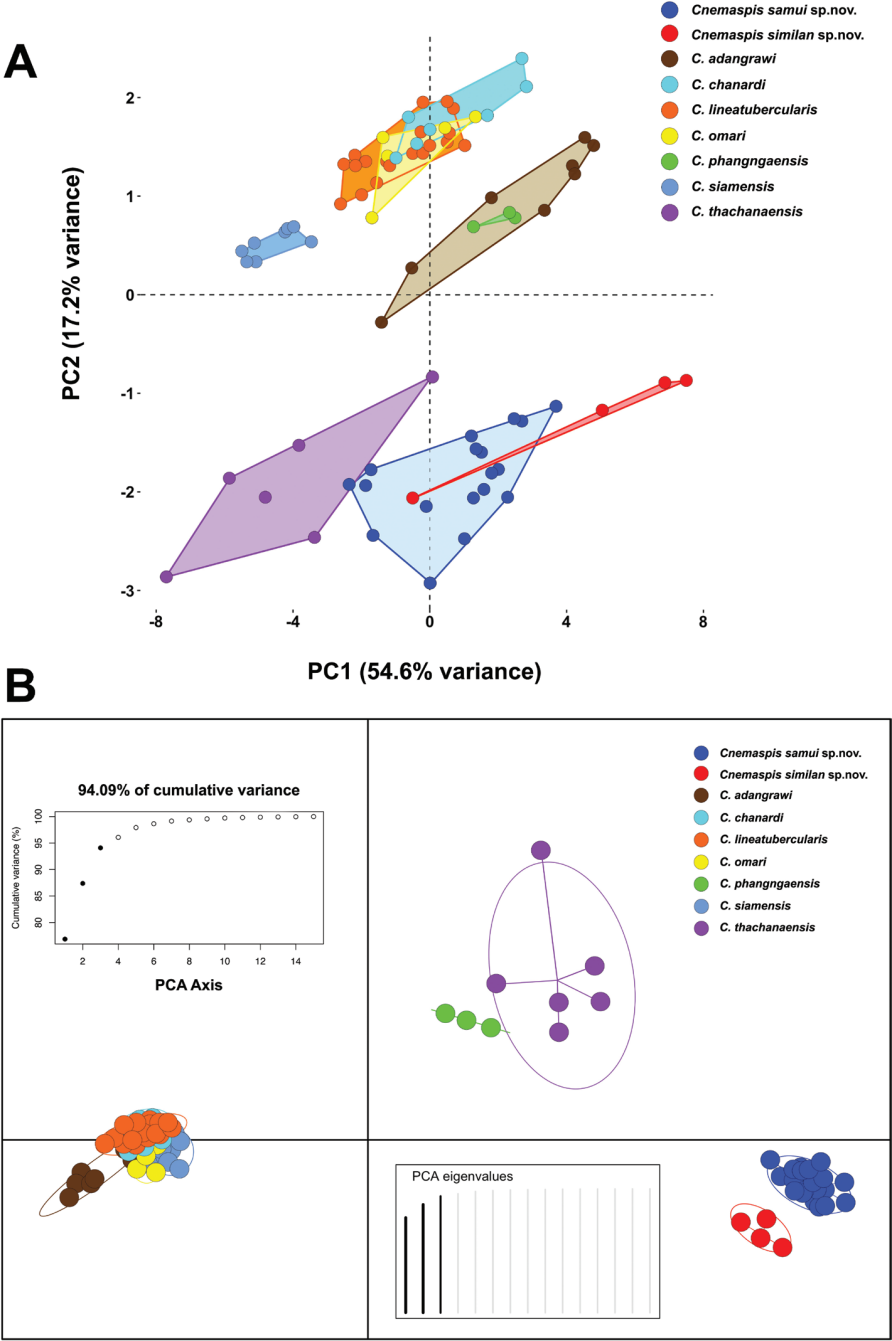
Multivariate analysis results of principal component analysis (PCA) and discriminant analysis of principal component (DAPC) of 14 morphological variables for nine OTUs (*N* = 78 individuals) of *Cnemaspis* in the *siamensis* group **A**PCA scatterplot showing morphospatial differentiation among nine species in the *siamensis* group **B**DAPC ordination of six PCs and discriminant eigenvalues showing morphospatial variation among nine species in the *siamensis* group.

Univariate analysis of variance (ANOVA) showed significant differences (*p* < 0.05) in morphometric characters among the Ko Samui population (OTU1), the Ko Similan population (OTU2), and seven congeners (OTU3–OTU9) in the *siamensis* group (Suppl. material 1: Table S1). These were also significantly different in the Tukey’s HSD pairwise tests (*p* < 0.05; Table 3). Multivariate analysis of PCA of nine species of *Cnemaspis* revealed morphological differences on a scatter plot of the first two components having eigenvalues > 1.0 (Fig. 2A). These first two components that accounted for 71.8% of the variation in the dataset showed that the Ko Samui and the Ko Similan samples clustered separately from seven congeners in the *siamensis* group (Table 4). The first principal component (PC1) accounted for 54.6% of the of variation and was most heavily loaded on five head characters (head length, head width, head depth, eye-ear distance, and eye-snout distance), two body characters (tibia length and axillar-groin length), and one tail character (tail width). The second principal components (PC2) accounted for an additional 17.2% of the variation and was heavily loaded on three head characters (internarial distance, interorbital distance, and ear length). Factor loadings of each component of 15 morphometric characteristics from nine OTUs of the *siamensis* group are provided in Table 4. The ordination of the first two components showed that the Ko Samui population overlapped with the Ko Similan population and *C.thachanaensis*. The DAPC (94.09% of cumulative variance) revealed the Ko Samui and the Ko Similan populations as distinct clusters, with general clustering of seven congeners in the *siamensis* group (Fig. 2B).

**Table 3.** Pairwise significant difference matrix from 15 size-corrected morphometric measurements of *Cnemaspissamui* sp. nov. and *Cnemaspissimilan* sp. nov. compared with seven congeners of the *Cnemaspissiamensis* group (Tukey’s HSD; *p*< 0.05). Measurement abbreviations are defined in the text.

No.	Species	1	2	3	4	5	6	7	8
1	*Cnemaspissamui* sp. nov.	–							
2	*Cnemaspissimilan* sp. nov.	SVL, FL, TBL, AG, HL, HW, IN, IO	–						
3	*C.adangrawi*	TBL, IN, IO	HW, IN, IO	–					
4	*C.chanardi*	FL, EL, IN, IO	SVL, TW, FL, HL, HW, IN, IO	FL, EL, IO	–				
5	*C.lineatubercularis*	TBL, HL, HW, EL, IN, IO	SVL, TW, FL, TBL, HL, HW, IN, IO	SVL, FL, TBL, HL, HW, EL, IO	TBL, HL, HW	–			
6	*C.omari*	TW, FL, TBL, HL, HW, EL, IN, IO	SVL, TW, HL, HW, IN, IO	TW, FL, HLHW, ES, EL, IO	FL, HW, IN	FL, TBL, IN	–		
7	*C.phangngaensis*	TW, TBL, IN	TW, IN	TW, IO	TW, FL	TW, FL, TBL, HL	HL, IN	–	
8	*C.siamensis*	SVL, TW, FL, TBL, HL, HW, ES, IN, IO	SVL, TW, FL, TBL, AG, HL, HW, ES, IN, IO	SVL, TW, FL, TBL, AG, HL, HW, ES, IN	SVL, TW, TBL, AG, HL, HW, ES, EL, IN, IO	SVL, TW, TBL, ES, IN, IO	SVL, FL, TBL, ES	SVL, FL, TBL, HL, ES, IN, IO	–
9	*C.thachanaensis*	SVL, TW, FL, TBL, AG, HL, HW, ES, EN, IN, IO	SVL, FL, TBL, AG, HL, HW, ES, EN, EL, IN	SVL, TW, FL, TBL, AG, HL, HW, ES, EN, IN, IO	SVL, TBL, AG, HL, HW, ES, EN, EL, IN, IO	SVL, TBL, AG, HW, ES, EN, EL, IN, IO	SVL, FL, TBL, AG, ES, EN, EL, IN	SVL, FL, TBL, AG, HL, HW, ES, EN, EL, IO	EN, EL, IN

**Table 4.** Summary of proportions of variance, standard deviation, eigenvalues and factor loadings from the 10 first principal components (PC) of 14 size-adjusted morphometric characters of two new insular species *Cnemaspissamui* sp. nov., *Cnemaspissimilan* sp. nov. and seven congeners of the *Cnemaspissiamensis* group including *C.adangrawi*, *C.chanardi*, *C.lineatubercularis*, *C.omari*, *C.phangngaensis*, *C.siamensis* and *C.thachanaensis*. Values highlighted in bold represent those with the greatest contribution (≥0.30). Measurement abbreviations are defined in the text.

Character	PC1	PC2	PC3	PC4	PC5	PC6	PC7	PC8	PC9	PC10
Proportion of Variance	54.6	17.2	6.2	5.7	5.1	4.3	2.3	1.3	1.0	0.8
Standard deviation	2.77	1.55	0.93	0.89	0.84	0.78	0.57	0.42	0.37	0.33
eigenvalues	7.65	2.406	0.872	0.793	0.711	0.606	0.32	0.179	0.137	0.112
TW	-0.293	-0.083	0.266	0.296	-0.093	0.017	-**0.747**	0.069	-0.054	0.169
FL	-0.263	-0.125	-**0.375**	-**0.362**	**0.414**	-0.207	-**0.317**	-0.01	0.005	**0.374**
TBL	-**0.308**	0.091	-0.217	-0.171	**0.401**	0.182	0.021	0.123	0.217	-**0.454**
AG	-**0.302**	0.233	-0.04	-0.132	-0.093	0.247	0.069	-**0.708**	-**0.358**	0.163
HL	-**0.324**	-0.118	-0.063	-0.020	0.037	**0.360**	0.229	**0.310**	0.064	**0.371**
HW	-**0.328**	-0.097	-0.009	-0.030	-0.115	**0.418**	0.100	0.016	-0.145	-0.267
HD	-**0.321**	-0.073	**0.332**	0.211	-0.114	0.066	0.175	0.170	-0.108	0.095
ED	-0.264	0.079	**0.422**	-0.232	0.237	-**0.518**	0.161	0.132	-**0.458**	-0.196
EE	-**0.323**	-0.005	-0.032	**0.422**	0.145	-0.073	0.080	0.071	0.209	-0.164
ES	-**0.304**	0.191	0.011	0.198	-0.086	-**0.370**	0.098	-**0.408**	**0.532**	-0.027
EN	-0.210	0.208	-**0.563**	0.093	-**0.500**	-**0.325**	0.119	**0.308**	-0.236	0.083
EL	-0.105	**0.439**	0.282	-**0.558**	-**0.364**	0.087	-0.158	0.214	**0.357**	0.004
IN	-0.096	-**0.565**	0.172	-0.231	-0.137	-0.155	**0.325**	-0.085	0.254	0.292
IO	0.113	**0.538**	0.14	0.211	**0.370**	0.079	0.236	0.131	0.032	**0.473**

### ﻿Taxonomic hypotheses

The Ko Samui and Ko Similan populations distinctly differed from all congeners in the *C.siamensis* group that were evaluated based on molecular analyses of mtDNA with high genetic distances, as well in the univariate analyses (ANOVA and Tukey’s HSD pairwise) and the multivariate analyses (PCA and DAPC) of morphology. Based on these corroborating lines of evidence, we hypothesize that the Ko Samui and the Ko Similan populations each represent new species, as described below.

### ﻿Systematics

#### ﻿ Cnemaspis samui sp. nov.

Taxon classificationAnimaliaSquamataGekkonidae

B963EB62-3B17-5468-B451-82D336208E45

https://zoobank.org/F75694D-7398-4084-BA37-21D5D1B40D03

**Figure 3.**
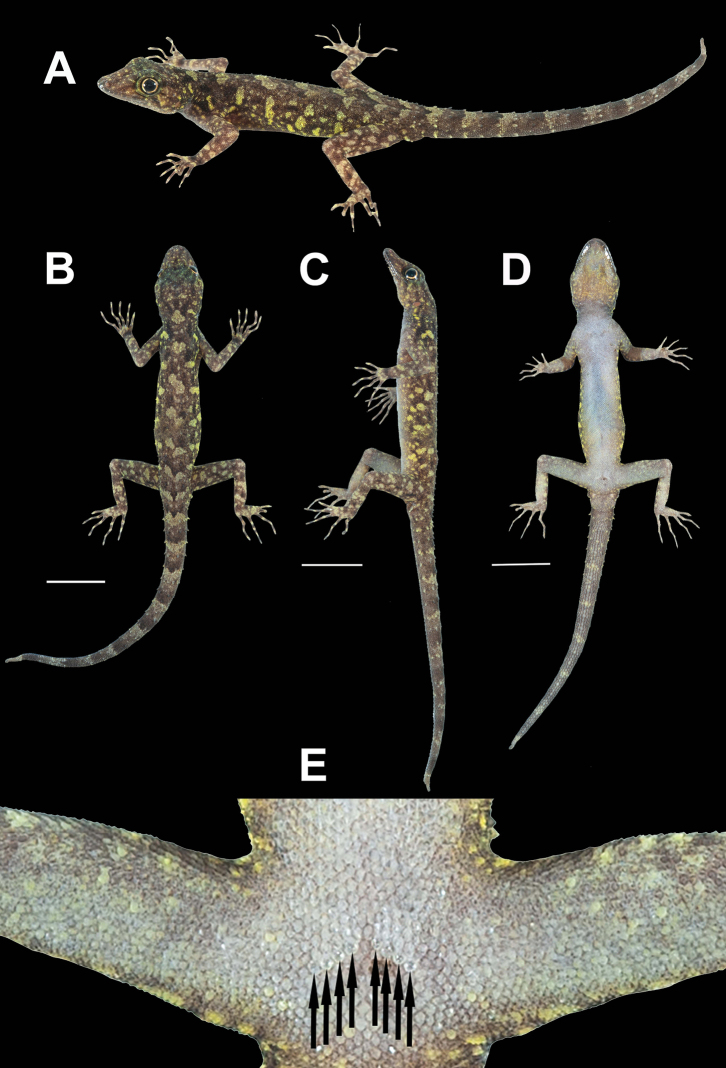
Adult male holotype of *Cnemaspissamui* sp. nov. (ZMKU R 00974) from Hin Lad Waterfall, Ko Samui, Ang Thong Subdistrict, Ko Samui District, Surat Thani Province, Thailand, in life **A** dorsolateral view **B** dorsal view **C** lateral view **D** ventral view **E** precloacal region showing distribution of pore-bearing scales (black arrows). Scale bars in dorsal, lateral, and ventral views: 10 mm.

**Figure 4.**
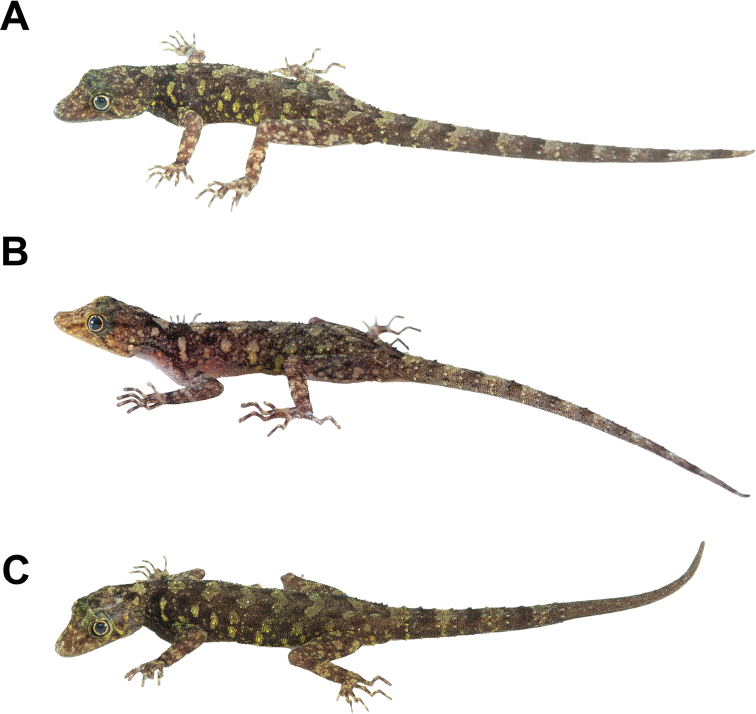
Coloration of *Cnemaspissamui* sp. nov. in dorsolateral view **A** adult male holotype ZMKU R 00974 **B** adult male paratype ZMKU R 00970 **C** adult male paratype ZMKU R 00971.

**Figure 5.**
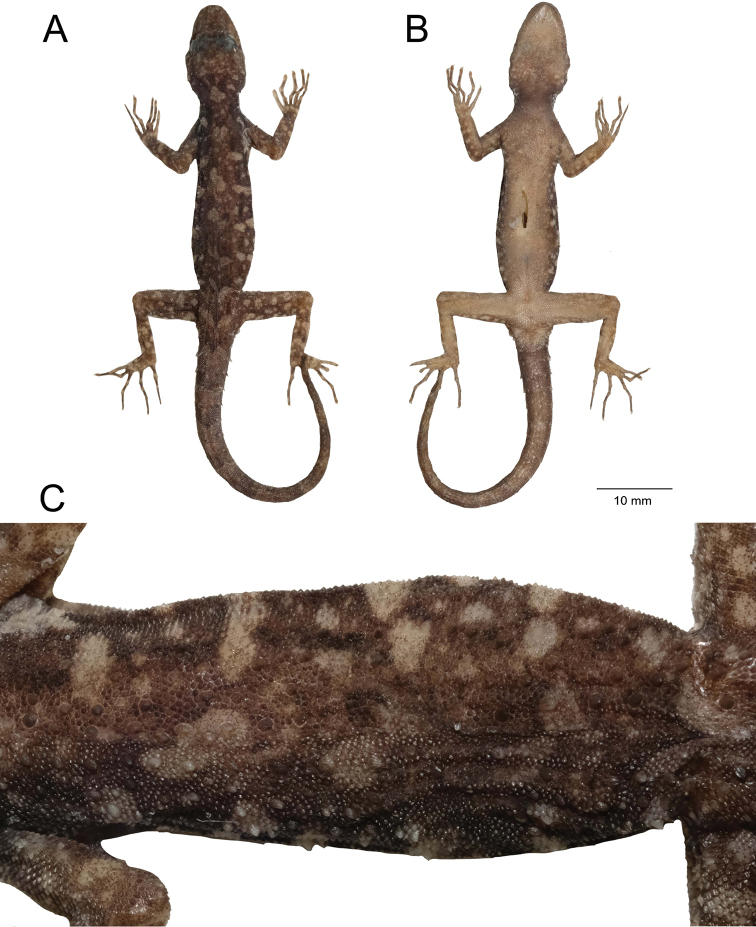
Adult male holotype of *Cnemaspissamui* sp. nov. (ZMKU R 00974) from Hin Lad Waterfall, Ko Samui, Ang Thong Subdistrict, Ko Samui District, Surat Thani Province, Thailand, in preservative. **A** dorsal view **B** ventral view **C** dorsal view of trunk. Scale bar in dorsal, and ventral views: 10 mm.

**Figure 6.**
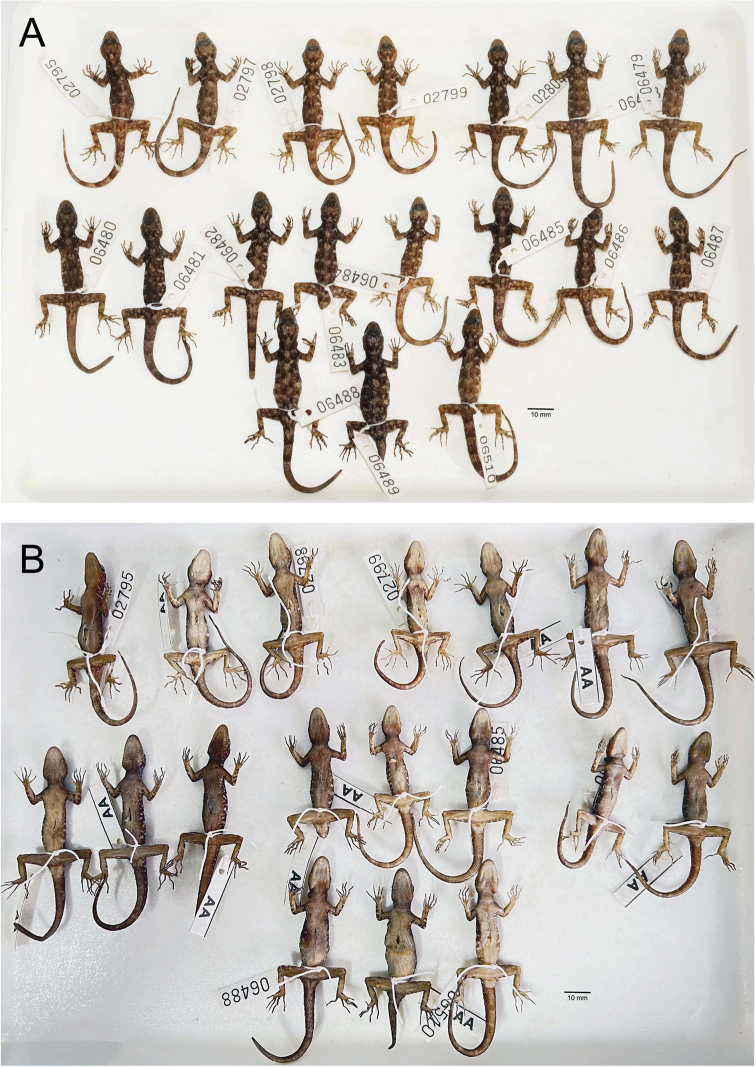
Paratypes of *Cnemaspissamui* sp. nov. in preservative. **A** dorsal view **B** ventral view; from left to right, top panel: ZMKU R 00966–00972; middle panel: ZMKU R 00973–00980; bottom panel: ZMKU R 00981–00983. Scale bars in dorsal and ventral views: 10 mm.

Figs 3 , 4 , 5 , 6 Ko Samui Rock Gecko Thai common name: Jing Jok Niew Yaow Ko Samui (จิ้งจกนิ้วยาวเกาะสมุย)

##### Holotype

**(Fig. 3).**ZMKU R 00974, adult male from Thailand, Surat Thani Province, Ko Samui District, Ang Thong Subdistrict, Hin Lad Waterfall (9°31.151'N, 99°57.598'E; 150 m a.s.l.), collected on 19 June 2018 by Natee Ampai, Attapol Rujirawan, Siriporn Yodthong and Korkhwan Termprayoon.

##### Paratypes

**(Fig. 4).** Seventeen paratypes (adult males = 14, adult females = 3). Five adult males (ZMKU R 00966–00970), same collection data as holotype except collected on 26 September 2015 by Natee Ampai, Attapol Rujirawan, Siriporn Yodthong, Korkhwan Termprayoon, and Anchalee Aowphol. Nine adult males (ZMKU R 00971–00973, ZMKU R 00975–00979 and ZMKU R 00983) and three adult females (ZMKU R 00980–00982), same data as holotype.

##### Diagnosis.

*Cnemaspissamui* sp. nov. differs from all other members of the *C.siamensis* group by having the following combination of characters: (1) SVL 37.0–42.3 mm in adult males (mean 39.90 ± 1.98 mm; *N* = 15) and 36.4–41.6 mm in adult females (mean 39.75 ± 2.91 mm; *N* = 3); (2) eight or nine supralabial and infralabial scales; (3) ventral scales keeled (4) 5–8 pore-bearing precloacal scales in males, arranged in a chevron, separated, pore rounded in males; (5) 25–27 paravertebral tubercles, arranged randomly; (6) 4–6 small, subconical spine-like tubercles present on lower flanks; (7) 22–25 subdigital lamellae under 4^th^ toe; (8) enlarged median subcaudal scale row present; (9) ventrolateral caudal tubercles anteriorly present; (10) one or two postcloacal tubercles on lateral surface of hemipenial swellings at the base of tail in males; and (11) gular region, abdomen, limbs and subcaudal region yellowish only in males.

##### Description of holotype.

An adult male in good state of preservation; 42.3 mm SVL; head relatively moderate in size (HL/SVL 0.27), narrow (HW/SVL 0.16), flattened (HD/HL 0.39), depressed (HD/SVL 0.11), and head distinct from neck; snout moderate (ES/HL 0.43), in lateral profile slightly concave; loreal region slightly inflated, canthus rostralis not prominent, smoothly rounded; postnasal region constricted medially; scales of rostrum round, juxtaposed, keeled, larger than conical scales on occiput; weak, supraorbital ridges; gular and throat scales granular, keeled and round; shallow frontorostral sulcus; eye large (ED/HL 0.21) with round pupil; orbit with extra-brillar fringe scales slightly largest anteriorly; scales on interorbitals and supercilium slightly keeled; eye to ear distance greater than eyes diameter (EE/ED 1.33); ear opening vertical, oval, taller than wide (EL/HL 0.09); rostral slightly concave; rostral bordered posteriorly by supranasals and internasal; rostral in contact laterally with first supralabials; 9R,L supralabials decreasing in size posteriorly; 8R,L infralabials decreasing in size posteriorly; nostril small, oval, oriented dorsoposteriorly, surrounded posteriorly by small postnasal scales; mental scales enlarged, subtriangular, concave, extending to level of second infralabials, bordered posteriorly by three large postmentals.

Body relatively slender, elongate (AG/SVL 0.42); small, keeled, dorsal scales equal in size throughout body intermixed with several large, keeled, scattered, conical tubercles; 26 paravertebral tubercles randomly arranged; four small, subconical spine-like tubercles on flanks; tubercles present on lower flanks; tubercles extend from occiput to tail; pectoral and abdominal scales keeled, round, flat, slightly larger than dorsal and not larger posteriorly; ventral scales of brachia smooth, raised and juxtaposed; eight separated pore-bearing precloacal scales, arranged in a chevron, with rounded pores; precloacal depression absent; femoral pores absent.

Fore and hind limbs moderately elongate, slender; scales beneath forearm slightly raised, smooth and subimbricate; subtibial scales keeled; palmar scales smooth, flat and subimbricate; digits long, slender, distinctly inflected joint with strong, slightly recurved claws; subdigital lamellae unnotched; lamellae beneath first phalanges wide; lamellae beneath phalanx immediately following inflection granular; lamellae of distal phalanges wide; lamellae beneath inflection large; interdigital webbing absent; enlarged submetatarsal scales on 1^st^ toe present; total subdigital lamellae on fingers I–V: 18-21-22-24-23 (right manus), 18-21-22-24-23 (left manus); fingers increase in length from first to fourth with fifth nearly equal in length as fourth; relative length of fingers IV>V>III>II>I; total subdigital lamellae on toes I–V: 14-20-21-24-23 (right pes), 14-(broken)-21-24-23 (left pes); toes increase in length from first to fourth with fifth nearly equal in length as fourth; relative length of toes IV>V>III>II>I.

Tail complete, entire cylindrical, relatively slender, swollen at the base; tail length (TL) 52.2 mm; tail length longer than snout-vent length (TL/SVL 1.23); subcaudal scales keeled, juxtaposed, larger than dorsal scales of the tail; shallow, middorsal furrow; deeper lateral caudal furrow present; enlarged, transverse caudal tubercles arranged in segmented whorls, encircling tail; enlarged median subcaudal scale row present; caudal tubercles present between upper and lower of lateral furrow; 1R,L enlarged postcloacal tubercle at lateral surface of hemipenial swellings at the base of tail.

##### Measurements of holotype

(in mm; Table 5). SVL 42.3; TL (complete tail) 52.2; TW 4.4; FL 6.5; TBL 7.9; AG 17.9; HL 11.5; HW 6.9; HD 4.5; ED 2.5; EE 3.3; ES 5.0; EN 4.0; EL 1.0; IN 1.1; IO 3.3.

**Table 5.** Descriptive measurements in millimeters and characters of the type series of *Cnemaspissamui* sp. nov. H = holotype; P = paratype; – = data unavailable or absent; C = complete; B = broken; R = regenerated. Measurement abbreviations are defined in the text.

Characters/Museum Number	ZMKU R 00974	ZMKU R 00966	ZMKU R 00967	ZMKU R 00968	ZMKU R 00969	ZMKU R 00970	ZMKU R 00971	ZMKU R 00972	ZMKU R 00973	ZMKU R 00975	ZMKU R 00976	ZMKU R 00977	ZMKU R 00978	ZMKU R 00979	ZMKU R 00983	ZMKU R 00980	ZMKU R 00981	ZMKU R 00982
Type series	H	P	P	P	P	P	P	P	P	P	P	P	P	P	P	P	P	P
Sex	Male	Male	Male	Male	Male	Male	Male	Male	Male	Male	Male	Male	Male	Male	Male	Female	Female	Female
SVL	42.3	40.1	41.2	40.8	37.0	38.7	41.7	41.5	40.7	41.0	40.4	35.6	40.1	36.7	40.6	36.4	41.2	41.6
Tail	C	C	C	R	B	C	R	C	R	B	B	C	C	C	C	C	R	R
TL	52.2	50.8	57.8	56.2	44.3	48.8	51.3	59.3	40.6	28.7	–	47.6	54.8	48.6	59.6	46.1	44.0	16.4
TW	4.4	3.9	3.8	4.0	3.9	4.0	4.2	4.2	4.1	4.3	4.4	3.6	4.1	3.7	4.1	3.6	4.2	3.9
FL	6.5	6.3	6.3	6.2	6.2	6.1	6.3	6.2	6.1	6.5	6.8	6.2	6.2	6.0	6.1	6.2	6.2	6.1
TBL	7.9	7.5	7.6	7.6	7.5	7.5	7.7	7.6	7.7	7.6	7.8	7.2	7.6	6.9	7.7	7.1	7.8	7.7
AG	17.9	17.7	17.7	17.7	16.1	16.1	17.7	17.6	17.7	17.7	17.6	15.5	17.6	16.1	17.8	15.2	17.9	17.9
HL	11.5	11.2	11.3	11.2	10.8	10.8	11.4	11.6	10.7	11.4	11.1	9.8	10.8	10.0	11.4	10.6	11.3	10.8
HW	6.9	6.6	6.9	6.8	6.6	6.6	6.8	6.8	6.8	6.8	6.7	6.2	6.7	6.2	6.9	6.2	6.9	6.8
HD	4.5	4.2	4.3	4.2	4.1	4.1	4.4	4.3	4.3	4.4	4.4	3.8	4.3	3.9	4.4	3.8	4.4	4.4
ED	2.5	2.3	2.4	2.2	2.2	2.3	2.4	2.4	2.1	2.2	2.2	2.3	2.2	2.2	2.4	2.2	2.4	2.4
EE	3.3	3.1	3.3	3.2	3.1	3.1	3.2	3.2	3.1	3.2	3.2	2.9	3.2	3.0	3.2	2.9	3.2	3.2
ES	5.0	4.9	4.9	4.9	4.5	4.6	4.8	4.8	4.7	4.9	4.9	4.6	4.9	4.7	4.8	4.5	4.9	4.9
EN	4.0	3.7	3.7	3.7	3.8	3.7	4.0	3.9	3.7	3.8	3.7	3.4	3.6	3.5	3.9	3.5	4.0	3.9
EL	1.0	0.9	0.8	0.8	0.9	0.7	0.9	1.0	1.0	0.9	0.9	0.9	0.8	0.9	0.9	0.9	0.9	0.8
IO	3.3	3.1	3.3	3.2	3.0	3.2	3.2	3.1	3.1	3.2	3.1	2.8	3.1	2.8	3.2	2.8	3.3	3.3
IN	1.1	1.0	1.1	1.0	0.9	1.0	1.1	1.0	1.0	1.0	1.0	1.0	0.9	1.0	1.1	1.0	1.0	1.1
Supralabial scales	9	9	9	9	9	9	8	9	8	9	9	9	9	9	9	9	9	9
Infralabial scales	8	9	8	8	8	8	8	8	8	9	8	8	8	8	8	8	8	8
No. of precloacal pores	8	7	8	5	6	8	7	8	7	6	7	8	7	5	7	–	–	–
Precloacal pore continuous (1) or separated (0)	0	0	0	0	0	0	0	0	0	0	0	0	0	0	0	–	–	–
Precloacal pores elongate (1) or round (0)	0	0	0	0	0	0	0	0	0	0	0	0	0	0	0	–	–	–
No. of paravertebral tubercles	26	26	25	27	26	27	27	26	27	25	27	26	25	27	27	27	27	26
Tubercles linearly arranged (1) or more random (0)	0	0	0	0	0	0	0	0	0	0	0	0	0	0	0	0	0	0
Tubercles present (1) or absent (0) on lower flanks	1	1	1	1	1	1	1	1	1	1	1	1	1	1	1	1	1	1
No. of 4^th^ toe lamellae	24	23	25	25	25	25	23	25	24	24	25	22	25	25	25	24	24	25
Lateral caudal furrows present (1) or absent (0)	1	1	1	1	1	1	1	1	1	1	1	1	1	1	1	1	1	1
Pectoral scales keeled (1) or smooth (0)	1	1	1	1	1	1	1	1	1	1	1	1	1	1	1	1	1	1
Ventral scales on thigh keeled (1) or smooth (0)	1	1	1	1	1	1	1	1	1	1	1	1	1	1	1	1	1	1
Subcaudal keeled (1) or smooth (0)	1	1	1	1	1	1	1	1	1	1	1	1	1	1	1	1	1	1
Subtibial scales keeled (1) or smooth (0)	1	1	1	1	1	1	1	1	1	1	1	1	1	1	1	1	1	1
Enlarged median subcaudal scale row (1) or not (0)	1	1	1	1	1	1	1	1	1	1	1	1	1	1	1	1	1	1
Caudal tubercles restricted to the single paravertebral row on each side (1) or not (0)	1	1	1	1	1	1	1	1	1	1	1	1	1	1	1	1	1	1

##### Coloration in life

**(Figs 3, 4A).** Dorsal ground color of head dark brown, top of head and snout bearing small, diffuse, finely speckled with yellowish spots; 3R,L thin, and faint dark postorbital stripes extending from eye to nape; pupil black with orange streak; irregular, pale yellowish marking on nape; a single yellowish prescapular crescent on shoulder each side, located at forelimb insertion dorsoanteriorly; dorsal ground color of body, limbs and tail brown overlain with black irregular blotches; two dark blotches form a bipartite pattern on nape; light-grey vertebral blotches extending from the nape to tail; flanks with scattered, incomplete light-grey to yellowish blotches becoming smaller posteriorly; tubercles on the whole body white or yellow; subconical spine-like yellowish tubercles on lower flanks; digits with dark brown and yellow bands; dorsum caudal bands light-grey and dark brown; ventral surfaces grayish-white intermixed with yellowish blotches on side of body; ventral pattern sexually dimorphic, gular, flanks, and caudal regions yellowish only in males; no dark markings on gular and belly; ventral side of caudal yellowish and indistinct bands.

##### Coloration in preservative

**(Figs 5, 6).** Dorsal ground color of head, body, limbs and tail darker brown than coloration in life; indistinct, irregular vertebral blotches; all yellowish spots and markings on head, body, limbs, and tail faded to whitish gray; banding on the tail faded and less prominent; ventral surface whitish gray with indistinct darker marking; gular, pectoral and tail regions with faint dark blotches.

##### Variation and additional information.

Most paratypes closely resemble the holotype in all aspect of pattern and coloration. Morphometric and meristic variation within the type series is presented in Table 5. Some paratypes differ in their degree of vertebral blotches. Sexual dimorphism in color pattern was apparent, as all adult male paratypes have yellowish coloration in the gular, flanks and caudal regions but this yellowish coloration was absent in females. ZMKU R 00968, ZMKU R 00971, ZMKU R 00973 (three adult males), and ZMKU R 00981–00982 (two adult females) have regenerated tails of uniform tan coloration. ZMKU R 00969 and ZMKU R 00975 (two adult males) have broken tail tips. ZMKU R 00976 (one adult male) has approximately three-fourth of the tail broken. ZMKU R 00966–00969, ZMKU R 00971, ZMKU R 00977, ZMKU R 00980, and ZMKU R 00983 (eight adult males) have paler dorsal markings that more resemble transverse bands than paravertebral blotches. ZMKU R 00969 and ZMKU R 00983 (two adult males) have 2R,1L enlarged postcloacal tubercles on the lateral surface of the hemipenial swelling at the base of tail.

##### Distribution.

**Figure 7.**
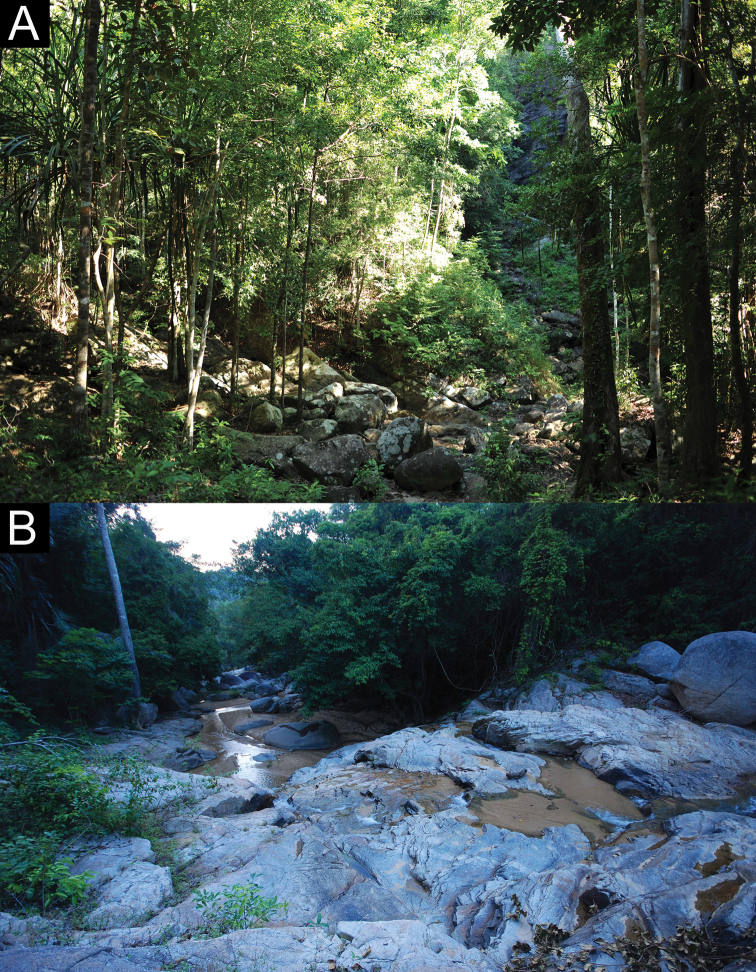
Habitats of *Cnemaspissamui* sp. nov. at the type locality **A** lowland evergreen forest with granitic outcrops **B** rocky stream outcrops along Lipa Yai Canal of Hin Lad Waterfall, Ko Samui, Ang Thong Subdistrict, Ko Samui District, Surat Thani Province, Thailand.

*Cnemaspissamui* sp. nov. is currently only known from the type locality at Hin Lad Waterfall (9°31.151'N, 99°57.598'E; 150 m a.s.l.; Fig. 7), Ang Thong Subdistrict, Ko Samui District, Surat Thani Province, Thailand, approximately 35 km off the mainland of Don Sak District, Surat Thani Province in the Gulf of Thailand.

##### Natural history.

The type locality is surrounded by lowland evergreen forest with granitic rocky outcrops along Lipa Yai Canal in the western part of Ko Samui. All specimens of *C.samui* sp. nov. were found along rocky stream outcrops of Hin Lad Waterfall during the day (1435–1752 h) and night (1800–1845 h) with air temperatures of 26.2–30.1 °C and relative humidity of 76.9–92.7%. Their microhabitats in rocky boulders were relatively dry and cool. The male holotype was found at night (1845 h) perched upside down on an overhanging surface of a granitic rock boulder near a stream. Most specimens were found on or within deep cracks or crevices of boulders, or in shaded areas of the boulder near a stream, except that ZMKU R 00969 was found on a tree trunk and ZMKU R 00977 was found in a soil hole at the base of a boulder. Two gravid females ZMKU R 00981–00982 were carrying one or two eggs in July 2018. Some juveniles (not collected) were mostly found perched on vegetation (e.g., log, vine, tree root). *Cnemaspissamui* sp. nov. is assumed to be a diurnal rock-dwelling species. During the day, geckos were found to be active, wary and fast-moving. They were most often observed clinging upside down to the undersides of rock boulders and within deep crevices. When disturbed, they would quickly move to deeper cover and hide in the shaded area between boulder and the ground. At night, they were found to be inactive, slow moving, sheltered in crevices or cracks on rock walls, or sleeping on vegetation near rock boulders, making them easier to approach than during the day. During field surveys, the larger nocturnal gekkonid *Cyrtodactyluszebraicus* (Taylor, 1962) was found in sympatry on the ground and vegetation near a stream.

##### Etymology.

The specific epithet *samui* is a noun in apposition and refers to the type locality of Ko Samui.

##### Comparisons.

*Cnemaspissamui* sp. nov. is distinguished from all members of the *siamensis* group (*C.adangrawi*, *C.chanardi*, *C.huaseesom*, *C.kamolnorranathi*, *C.lineatubercularis*, *C.omari*, *C.phangngaensis*, *C.punctatonuchalis*, *C.selenolagus*, *C.siamensis*, *C.thachanaensis*, and *C.vandeventeri*) by having a unique combination of morphological characteristics (Table 6) and uncorrected pairwise sequence divergences of mtDNA (ND2) of 8.86–26.83% (Table 2).

**Table 6.** Meristic character state and color pattern of species in the *Cnemaspissiamensis* group. SVL taken in millimeters and measurement abbreviations are defined in the text. – = data unavailable, w = weak.

Characters / Species	*Cnemaspissamui* sp. nov.	*Cnemaspissimilan* sp. nov.	*C.adangrawi*	*C.chanardi*	*C.huaseesom*	*C.kamolnorranathi*	*C.lineatubercularis*	*C.omari*	*C.phangngaensis*	*C.punctatonuchalis*	*C.selenolagus*	*C.siamensis*	*C.thachanaensis*	*C.vandeventeri*
Sample size	18	4	15	25	5	4	19	8	2	5	2	12	6	3
Maximum SVL	42.3	48.1	44.9	40.9	43.5	37.8	41.8	41.3	42.0	49.6	36.2	39.7	39.0	44.7
Supralabial scales	8 or 9	8 or 9	10	8–10	7–10	8 or 9	9	8 or 9	10	8	10 or 11	8 or 9	10 or 11	8 or 9
Infralabial scales	8 or 9	7 or 8	9	8	6–9	7 or 8	9	7 or 8	10	7 or 8	10	6–8	9–11	7–9
No. of pore-bearing precloacal scales	5–8	1	6–8	6–8	5–8	6 or 7	4–7	3–6	4	0	6 or 7	0	0	4
Pore-bearing precloacal scales row continuous (1) or separated (0)	0	–	0	0	1	1	0	0	1	–	1	–	–	0
Pore-bearing precloacal scales elongate (1) or round (0) shapes	0	0	0	0	0	1	0	0	0	–	1	–	–	0
No. of paravertebral tubercles	25–27	24 or 25	23–25	22–25	18–24	19–24	19–21	22–29	22	24–27	16–18	19–25	15–19	25–29
Paravertebral tubercles linearly arranged (1) or more random (0)	0	0	0	0	w or 0	w	1	w or 0	1	w	0	0	1	0
Tubercles present (1) or absent (0) on lower flanks	1	1	0	1	1	1	1	w or 1	0	1	0	1	1	1
No. of 4^th^ toe lamellae	22–25	23 or 24	26–28	26–29	21–31	24–28	27–29	25–28	29	29–31	22	24–26	24	24–28
Ventral scales keeled (1) or smooth (0)	1	1	1	1	0	w or 0	1	1	1	0	0	1	1	1
Subcaudal scales keeled (1) or smooth (0)	1	1	1	1	0	1	1	1	1	0	0	1	1	1
Single median row of keeled subcaudals (1) or smooth (0) scales	1	1	1	0	0	w	1	0	1	0	–	0	1	w
Enlarged median subcaudal scales row (1) or not (0)	1	0	0	1	0	w	0	0	0	1	0	1	0	1
Caudal tubercles restricted to a single paravertebral row on each side (1) or not (0)	0	0	0	0	0	0	1	0	1	0	–	0	1	0
Ventrolateral caudal tubercles anteriorly present (1) or not (0)	1	1	1	0	0	0	1	0	1	1	0	0	1	0
No. of postcloacal tubercles in males	1 or 2	2	1	1	1 or 2	1 or 2	1	1	2	1–3	2	1 or 2	0	1–3
Subtibial scales keeled (1) or smooth (0)	1	1	1	1	0	0 or 1	1	1	1	1	0	1	1	1
Yellow coloration in the subcaudal region present (1) or not (0)	1	0	0	1	1	0	1	1	1	0	0	0	0	0
Ventral pattern sexually dimorphic present (1) or not (0)	1	1	1	1	1	0	1	1	1	1	–	1	1	1

*Cnemaspissamui* sp. nov. is distinguished from *C.adangrawi*Ampai et al. 2019 by having maximum SVL of 42.3 mm (vs. 44.9 mm); eight or nine supralabial scales (vs. 10 scales); tubercles on lower flanks present (vs. absent); 22–25 lamellae under 4^th^ toe (vs. 26–28 lamellae); enlarged median row of subcaudal scales present (vs. absent); and yellow coloration in the subcaudal region present (vs. absent).

*Cnemaspissamui* sp. nov. is distinguished from *C.chanardi*Grismer et al. 2010 by having maximum SVL 42.3 mm (vs. 40.9 mm); 22–25 lamellae under 4^th^ toe (vs. 26–29 lamellae); single median row of subcaudals keeled (vs. smooth); and ventrolateral caudal tubercles anteriorly present (vs. absent).

*Cnemaspissamui* sp. nov. is distinguished from *C.huaseesom*Grismer et al. 2010 by having maximum SVL of 42.3 mm (vs. 43.5 mm); pore-bearing precloacal scales row separated (vs. continuous); 25–27 paravertebral tubercles (vs. 18–24 tubercles); ventral and subcaudal scales keeled (vs. smooth); single median row of subcaudals keeled (vs. smooth); enlarged median row of subcaudal scales present (vs. absent); ventrolateral caudal tubercles anteriorly present (vs. absent); and subtibial scales keeled (vs. smooth).

*Cnemaspissamui* sp. nov. is distinguished from *C.kamolnorranathi*Grismer et al. 2010 by having maximum SVL 42.3 mm (vs. 37.8 mm); pore-bearing precloacal scales row separated (vs. continuous); pore-bearing precloacal scales rounded (vs. elongated); 25–27 paravertebral tubercles (vs. 19–24 tubercles); enlarged median subcaudal scale row present (vs. absent); ventrolateral caudal tubercles anteriorly present (vs. absent); yellow coloration in the subcaudal region present (vs. absent); and ventral pattern sexually dimorphism present (vs. absent).

*Cnemaspissamui* sp. nov. is distinguished from *C.lineatubercularis*Ampai et al. 2020 by having maximum SVL 42.3 mm (vs. 41.8 mm); 25–27 paravertebral tubercles (vs. 19–21 tubercles); paravertebral tubercles randomly arranged (vs. linearly arranged); 22–25 lamellae under 4^th^ toe (vs. 27–29 lamellae); enlarged median row of subcaudal scales present (vs. absent); and caudal tubercles restricted to a single paravertebral row on each side absent (vs. present).

*Cnemaspissamui* sp. nov. is distinguished from *C.omari*Grismer et al. 2014 by having maximum SVL 42.3 mm (vs. 41.3 mm); single median row of subcaudals keeled (vs. smooth); enlarged median row of subcaudal scales present (vs. absent); and ventrolateral caudal tubercles anteriorly present (vs. absent).

*Cnemaspissamui* sp. nov. is distinguished from *C.phangngaensis*Wood et al. 2017 by having eight or nine supralabial scales (vs. 10 scales); eight or nine infralabial scales (vs. 10 scales); 5–8 pore-bearing precloacal scales in males (vs. four scales); pore-bearing precloacal scales row separated (vs. continuous); 25–27 paravertebral tubercles (vs. 22 tubercles); paravertebral tubercles randomly arranged (vs. linearly arranged); tubercles on lower flanks present (vs. absent); 22–25 lamellae under 4^th^ toe (vs. 29 lamellae); enlarged median row of subcaudal scales present (vs. absent); and caudal tubercles restricted to a single paravertebral row on each side absent (vs. present).

*Cnemaspissamui* sp. nov. is distinguished from *C.punctatonuchalis*Grismer et al. 2010 by having maximum SVL of 42.3 mm (vs. 49.6 mm); pore-bearing precloacal scales present (vs. absent); 22–25 lamellae under 4^th^ toe (vs. 29–31 lamellae); ventral and subcaudal scales keeled (vs. smooth); single median row of subcaudals keeled (vs. smooth); and yellow coloration in the subcaudal region present (vs. absent).

*Cnemaspissamui* sp. nov. is distinguished from *C.selenolagus*Grismer et al. 2020 by having maximum SVL 42.3 mm (vs. 36.2 mm); eight or nine supralabial scales (vs. 10 or 11 scales); eight or nine infralabial scales (vs. 10 scales); pore-bearing precloacal scales row separated (vs. continuous); pore-bearing precloacal scales shape rounded (vs. elongated); 25–27 paravertebral tubercles (vs. 16–18 tubercles); tubercles on lower flanks present (vs. absent); enlarged median row of subcaudal scales present (vs. absent); ventrolateral caudal tubercles anteriorly present (vs. absent); subtibial scales keeled (vs. smooth); and yellow coloration in the subcaudal region present (vs. absent).

*Cnemaspissamui* sp. nov. is distinguished from *C.siamensis* (Smith, 1925) by having maximum SVL 42.3 mm (vs. 39.7 mm); pore-bearing precloacal scales present (vs. absent); single median row of subcaudals keeled (vs. smooth); ventrolateral caudal tubercles anteriorly present (vs. absent); and yellow coloration in the subcaudal region present (vs. absent).

*Cnemaspissamui* sp. nov. is distinguished from *C.thachanaensis*Wood et al. 2017 by having maximum SVL 42.3 mm (vs. 39.0 mm); eight or nine supralabial scales (vs. 10 or 11 scales); pore-bearing precloacal scales present (vs. absent); 25–27 paravertebral tubercles (vs. 15–19 tubercles); paravertebral tubercles randomly arranged (vs. linearly arranged); enlarged median row of subcaudal scales present (vs. absent); caudal tubercles restricted to a single paravertebral row on each side absent (vs. present); one or two postcloacal tubercles in males (vs. absent); and yellow coloration in the subcaudal region present (vs. absent).

*Cnemaspissamui* sp. nov. is distinguished from *C.vandeventeri*Grismer et al. 2010 by having maximum SVL of 42.3 mm (vs. 44.7 mm); 5–8 pore-bearing precloacal scales (vs. four scales); ventrolateral caudal tubercles anteriorly present (vs. absent); and having yellow coloration in the subcaudal region present (vs. absent).

#### ﻿ Cnemaspis similan sp. nov.

Taxon classificationAnimaliaSquamataGekkonidae

FDCA0555-16B3-52E5-92A7-73D90ADA47E3

https://zoobank.org/AF6821E3-D520-40D3-A076-EA96CFCAB6E3

**Figure 8.**
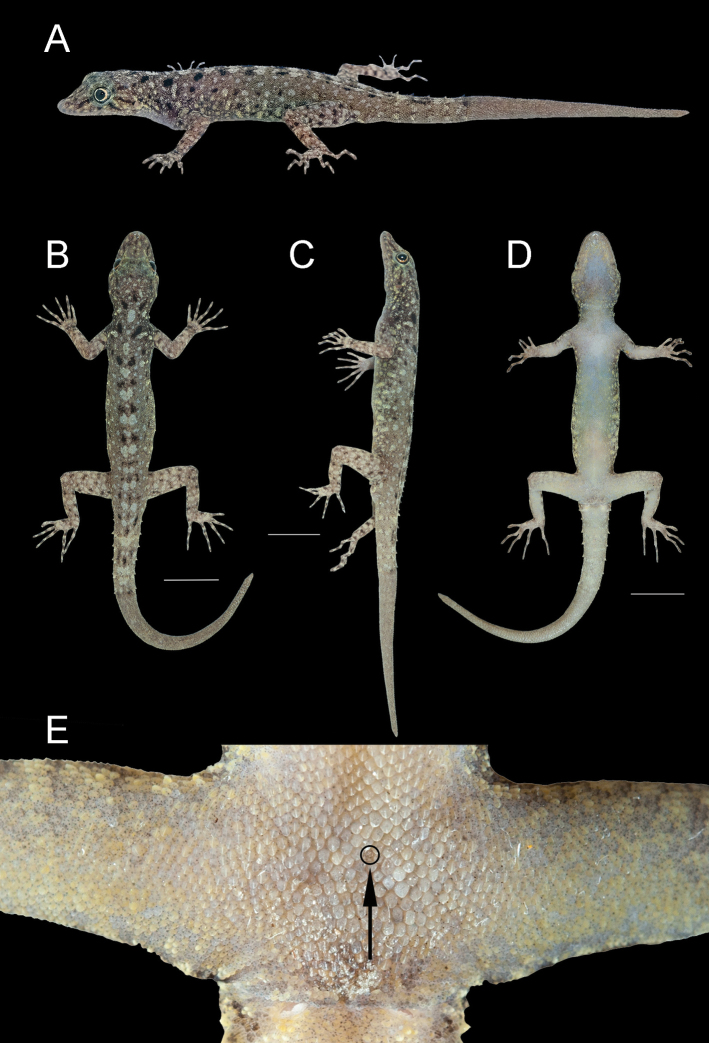
Adult male holotype of *Cnemaspissimilan* sp. nov. (ZMKU R 00984) from Ao Nguang Chang Bay, Ko Similan, Mu Ko Similan National Park, Lam Kaen Subdistrict, Thai Mueang District, Phang-nga Province, Thailand, in life **A** dorsolateral view **B** dorsal view **C** lateral view **D** ventral view **E** precloacal region showing distribution of pore-bearing scale (black arrow). Scale bars in dorsal, lateral, and ventral views: 10 mm.

**Figure 9.**
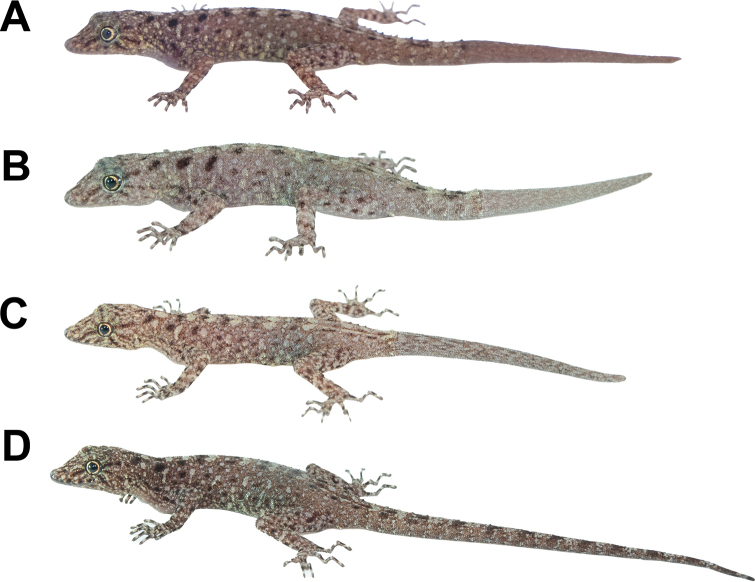
Coloration of adult *Cnemaspissimilan* sp. nov. in dorsolateral view **A** adult male holotype ZMKU R 00986 **B** adult female paratype ZMKU R 00985 **C** adult female paratype ZMKU R 00986 **D** adult female paratype ZMKU R 00987.

**Figure 10.**
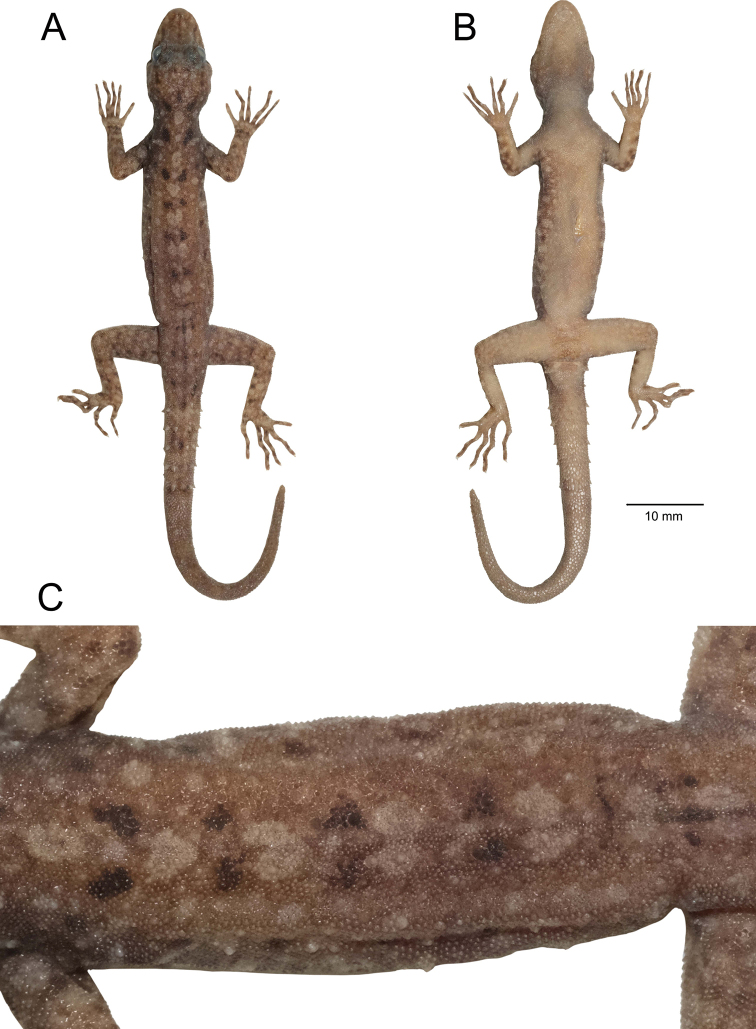
Adult male holotype of *Cnemaspissimilan* sp. nov. (ZMKU R 00984) from Ao Nguang Chang Bay, Ko Similan, Mu Ko Similan National Park, Lam Kaen Subdistrict, Thai Mueang District, Phang-nga Province, Thailand, in preservative **A** dorsal view **B** ventral view **C** dorsal view of trunk. Scale bar in dorsal and ventral views: 10 mm.

**Figure 11.**
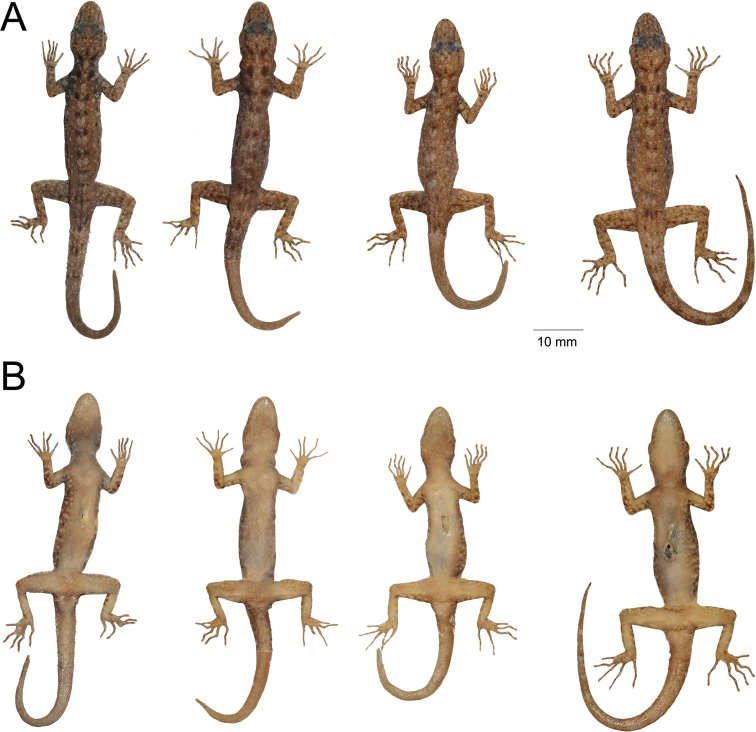
*Cnemaspissimilan* sp. nov. in preservative **A** dorsal view (top panel) **B** ventral view (bottom panel); from left to right: ZMKU R 00984–00987. Scale bar in dorsal and ventral views: 10 mm.

Figs 8 , 9 , 10 , 11 Ko Similan Rock Gecko Thai common name: Jing Jok Niew Yaow Ko Similan (จิ้งจกนิ้วยาวเกาะสิมิลัน)

##### Holotype

**(Fig. 8).**ZMKU R 00984, adult male from Thailand, Phang-nga Province, Thai Mueang District, Lam Kaen Subdistrict, Mu Ko Similan National Park, Ko Similan, Ao Nguang Chang Bay (8°64.840'N, 97°64.834'E; 13 m a.s.l.), collected on 5 March 2018 by Natee Ampai, Attapol Rujirawan, Siriporn Yodthong and Piyawan Puanprapai.

##### Paratypes

**(Fig. 9).** Three adult females paratypes. ZMKU R 00985–00986 (two adult females), same data as holotype. ZMKU R 00987 (one adult female), same data as holotype except collected on 6 March 2018 by Natee Ampai, Attapol Rujirawan, Siriporn Yodthong and Piyawan Puanprapai.

##### Diagnosis.

*Cnemaspissimilan* sp. nov. can be distinguished from all other members of the *C.siamensis* group by having the following combination of characters: (1) SVL of 47.6 mm in adult male and 38.6–48.1 mm (mean 43.6 ± 4.8 mm, *N* = 3) in adult females; (2) eight or nine supralabial and seven or eight infralabial scales; (3) ventral scales keeled (4) one pore-bearing precloacal scale, pore rounded in male; (5) 24 or 25 paravertebral tubercles, arranged randomly; (6) five small, elongated, spine-like tubercles on lower flanks; (7) 23 or 24 subdigital lamellae under the 4^th^ toe; (8) no enlarged median subcaudal scale row; (9) ventrolateral caudal tubercles anteriorly present; (10) two postcloacal tubercles on lateral surface of hemipenial swellings at tail base in male; (11) sexual dimorphism in dorsal and ventral patterns; and (12) pale yellow reticulum on head, neck, flanks, belly and limbs only in male.

##### Description of holotype.

An adult male in good state of preservation; 47.6 mm SVL; head moderate in size (HL/SVL 0.26), narrow (HW/SVL 0.16), flattened (HD/HL 0.39) and head distinct from neck; snout moderate (ES/HL 0.43), in lateral profile concave; loreal region marginally inflated, canthus rostralis nearly absent; postnasal region concave medially; scales of rostrum smooth, raised, larger than conical scales on occiput; weak and faint supraorbital ridges; gular scales granular, keeled, rounded, juxtaposed; throat scales granular, keeled, flat, subimbricate; shallow frontonasal sulcus; eye large (ED/HL 0.19); pupil round; extra-brillar fringe scales small in general but slightly larger anteriorly; scales on interorbitals and supercilium keeled; eye to ear distance greater than eyes diameter (EE/ED 1.50); ear opening elongate, much taller than wide (EL/HL 0.08); rostral concave dorsally; rostral bordered posteriorly by supranasals and laterally by first supralabials; 8R,L supralabials decreasing in size posteriorly; 7R,L infralabials decreasing in size posteriorly; nostril small, elliptical, oriented dorsoposteriorly, bordered posteriorly by small postnasal scales; mental scales large, triangular, flat, extending to level of second infralabial scales, bordered posteriorly by three large postmental scales.

Body robust, not elongate (AG/SVL 0.41); small, raised, keeled, dorsal scales equal in size throughout body intermixed with numerous large, keeled, multicarinate tubercles; 24 paravertebral tubercles randomly arranged; five small, elongated, spine-like tubercles on flanks; tubercles present on lower flanks; tubercles extend from occiput to tail; pectoral and abdominal scales keeled, round, flat, imbricate; abdominal scales larger than pectoral and dorsal scales; ventral scales of brachia smooth, raised and juxtaposed; one pore-bearing precloacal scale, with rounded pore; precloacal depression absent; femoral pores absent.

Fore and hind limbs moderately long, slender; scales beneath forearm slightly raised, smooth and subimbricate; subtibial scales keeled; palmar scales keeled, flat and subimbricate; digits long, slender with inflected joint; claws slightly recurved; subdigital lamellae unnotched; lamellae beneath first phalanges wide; lamellae beneath phalanx immediately following inflection granular; lamellae of distal phalanges wide; lamellae beneath inflection large; interdigital webbing generally absent; enlarged submetatarsal scales on 1^st^ toe present; total subdigital lamellae on fingers I–V: 15-21-22-24-23 (right manus), 15-21-23-24-23 (left manus); fingers increase in length from first to fourth with fifth nearly equal in length as fourth; relative length of fingers IV>V>III>II>I; total subdigital lamellae on toes I–V: 17-20-22-24-23 (right pes), 17-19-22-24-23 (left pes); toes increase in length from first to fourth with fifth nearly equal in length as fourth; relative length of toes IV>V>III>II>I.

Tail regenerated, subcylindrical, relatively swollen at the base; tail length (TL) 49.6 mm; tail length longer than head and body (TL/SVL 1.04); dorsal and ventral scales at the tail base similar in size on mid-body dorsum; subcaudal scales keeled, juxtaposed, larger than dorsal scale of the tail size; shallow, middorsal furrow; lateral caudal furrow present; enlarged, transverse caudal tubercles arranged in segmented whorls, encircling tail; enlarged median subcaudal scale row absent; caudal tubercles present between upper and lower of lateral furrow; rest of the tail regenerated, slightly keeled, imbricate scales with no enlarged tubercles; scales on ventral aspect of the regenerated tail marginally larger in size than mid-body ventrals; 2R,L enlarge postcloacal tubercle at lateral surface of hemipenial swellings at the tail base.

##### Measurements of holotype

(in mm; Table 7). SVL 47.6; TL (regenerated tail) 49.6; TW 4.6; FL 6.8; TBL 8.6; AG 19.6; HL 12.4; HW 7.8; HD 4.8; ED 2.4; EE 3.6; ES 5.3; EN 4.0; EL 1.0; IN 1.1; IO 3.1.

**Table 7.** Descriptive measurements in millimeters and characters of the type series of *Cnemaspissimilan* sp. nov. H = holotype; P = paratype; – = data unavailable or absent; C = complete; R = regenerated. Measurement abbreviations are defined in the text.

Characters / Museum number	ZMKU R 00984	ZMKU R 00985	ZMKU R 00986	ZMKU R 00987
Sex	Male	Female	Female	Female
Type series	H	P	P	P
SVL	47.6	48.1	38.6	44.2
Tail	R	R	R	C
TL	49.6	43.2	37.6	58.2
TW	4.6	4.6	4.1	4.4
FL	6.8	6.9	6.2	6.6
TBL	8.6	8.8	7.5	8.4
AG	19.6	19.8	16.6	19.4
HL	12.4	12.6	10.4	12.1
HW	7.8	7.9	6.5	7.7
HD	4.8	4.9	4.1	4.6
ED	2.4	2.4	2.1	2.3
EE	3.6	3.7	3.0	3.4
ES	5.3	5.4	4.3	4.9
EN	4.0	4.1	3.4	3.7
EL	1.0	1.0	0.9	1.0
IO	3.1	3.2	2.6	2.6
IN	1.1	1.1	0.9	1.0
Supralabial scales	8	9	9	9
Infralabial scales	7	8	8	8
No. of precloacal pores	1	–	–	–
Precloacal pore continuous (1) or separated (0)	–	–	–	–
Precloacal pores elongate (1) or round (0)	0	–	–	–
No. of paravertebral tubercles	24	25	25	24
Tubercles linearly arranged (1) or more random (0)	0	0	0	0
Tubercles present (1) or absent (0) on lower flanks	1	1	1	1
No. of 4^th^ toe lamellae	24	24	23	23
Lateral caudal furrows present (1) or absent (0)	1	1	1	1
Pectoral scales keeled (1) or smooth (0)	1	1	1	1
Ventral scales on thigh keeled (1) or smooth (0)	1	1	1	1
Subcaudal keeled (1) or smooth (0)	1	1	1	1
Subtibial scales keeled (1) or smooth (0)	1	1	1	1
Enlarged median subcaudal scale row (1) or not (0)	0	0	0	0
Caudal tubercles restricted to the single paravertebral row on each side (1) or not (0)	1	1	1	1

##### Coloration in life

**(Figs 8, 9).** Dorsal ground color of head brown, top of head and snout bearing diffuse, mottled with smaller yellowish markings; 3R,L vertical, thin and fine dark stripes extending from postorbital to neck; 1R,L indistinct darker stripes runs from preorbital to supranasal; pupil black with orange streak; irregular, faint pale yellow reticulum on lateral surface of head, neck and flanks; 1R,L light-colored prescapular crescent on shoulder, located at forelimb insertion dorsoanteriorly; two dark streaks form a bipartite pattern on neck; dorsal ground color of body and tail brown with irregular black blotches except much paler brown on limbs; pale sage vertebral blotches run from the nape to tail; flanks with smaller dark and larger pale yellow streaks; enlarged conical spine-like yellowish tubercles on lower flanks; tubercles on the whole body pale sage and pale yellow; digits with distinct dark and pale bands; dorsum of limbs pale brown with dark blotches randomly arranged; ventral surfaces pale greyish intermixed with pale yellowish blotches on gular, neck, limbs and belly; no markings on gular and belly regions; original part of the tail brown with dark streaks form a bipartite pattern; regenerated part of the tail brown without bands; ventral side of tail pale greyish with no markings.

##### Coloration in preservative

**(Figs 10, 11).** Overall coloration of head, body, limbs, flanks and tail about the same as in life. Dorsal ground color of the whole-body became faded. The pale tones of limbs and tail darker than in life. Vertebral blotches run from the nape to tail became paler than in life. All pale yellowish coloration on head, limbs, flanks fade to creamy white. Ventral region of the whole-body homogenously tan colored.

##### Variation and additional information.

Due to having only a single adult male (*N* = 1), variation in adult males is currently unknown. Most paratypes approximate the holotype in general features of body pattern and coloration. Adult females lack pore-bearing precloacal scale. Pale yellowish markings in head, neck, limbs, flanks and caudal regions were also absent in adult females. Three adult females have paler dorsal markings than the holotype. ZMKU R 00985 and ZMKU R 00986 have regenerated tails of uniform tan colored. ZMKU R 00985 has a large calcium sac on each side of the neck. ZMKU R 00985 has also broken left 4^th^ pes.

##### Distribution.

**Figure 12.**
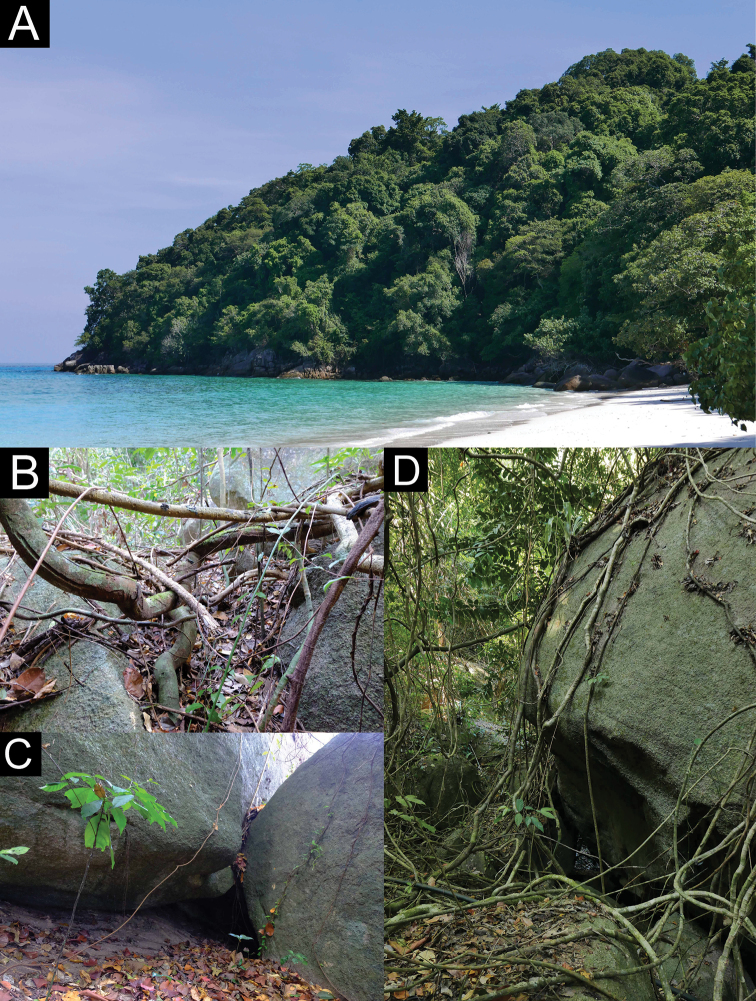
Habitats of *Cnemaspissimilan* sp. nov. at the type locality **A** mixed evergreen forest with shrub and beach forests **B** microhabitat of holotype on tree near granitic rock boulder **C** microhabitat of paratypes in granitic rock boulder **D** microhabitat of paratypes in rock wall with vegetations (tree trunk, root or vine) of Ao Nguang Chang Bay, Ko Similan, Mu Ko Similan National Park, Lam Kaen Subdistrict, Thai Mueang District, Phang-nga Province, Thailand.

*Cnemaspissimilan* sp. nov. is known only from the type locality at Ao Nguang Chang Bay (8°64.840'N, 97°64.834'E; 13 m a.s.l.; Fig. 12), Ko Similan, Lam Kaen Subdistrict, Thai Mueang District, Phang-nga Province, Thailand, approximately 65 km off the mainland of Thai Mueang District, Phang-nga Province in the Andaman Sea.

##### Natural history.

The type locality is dominated by mixed evergreen forest with shrub and beach forests. Ao Nguang Chang Bay is located at the southern part of the largest island, Ko Similan (= Ko Pad). All specimens of *C.similan* sp. nov. were found in granitic rocky outcrops near Ao Nguang Chang Bay during the day (1542 h) and night (2023–2049 h) with an air temperature of 28.4 °C and relative humidity of 86%. Granitic boulder surfaces appeared to be relatively dry and cool. The male holotype was found during the night (2023 h) on a tree near a boulder. Most paratypes (ZMKU R 00985–00986) were found during the day time on vegetation (tree trunks, roots, or vines) except ZMKU R 00987, which was perched on a rock wall. *Cnemaspissimilan* sp. nov. seems to be a diurnal rock-dwelling species. During the day, geckos were generally active, quite wary and quickly retreated when approached or disturbed. At night, geckos were found inactive or sleeping on vegetation near crevices or cracks of rock boulder as high as 2 m above the ground. They were often found clinging upside down to the underside of rock boulder overhang. During field surveys, the larger, nocturnal gekkonid *Cyrtodactylusoldhami* (Theobald, 1876) was found in sympatry on the ground and vegetation near boulders.

##### Etymology.

The specific epithet *similan* is a noun in apposition and refers to the type locality of Ko Similan.

##### Comparisons.

*Cnemaspissimilan* sp. nov. can be distinguished from 13 congeners of the *siamensis* group (*C.adangrawi*, *C.chanardi*, *C.huaseesom*, *C.kamolnorranathi*, *C.lineatubercularis*, *C.omari*, *C.phangngaensis*, *C.punctatonuchalis*, *C.samui* sp. nov., *C.selenolagus*, *C.siamensis*, *C.thachanaensis*, and *C.vandeventeri*) by having a unique combination of morphological characters (Table 6) and uncorrected pairwise sequence divergences in mtDNA (ND2) of 8.16–27.11% (Table 2).

*Cnemaspissimilan* sp. nov. is distinguished from *C.adangrawi*Ampai et al. 2019 by having maximum SVL 48.1 mm (vs. 44.9 mm); seven or eight infralabial scales (vs. nine scales); one pore-bearing precloacal scale (vs. 6–8 scales); eight or nine supralabial scales (vs. 10 scales); tubercles on lower flanks present (vs. absent); 23 or 24 lamellae under 4^th^ toe (vs. 26–28 lamellae); and two postcloacal tubercles in males (vs. one tubercle).

*Cnemaspissimilan* sp. nov. is distinguished from *C.chanardi*Grismer et al. 2010 by having maximum SVL 48.1 mm (vs. 40.9 mm); one pore-bearing precloacal scale (vs. 6–8 scales); 23 or 24 lamellae under 4^th^ toe (vs. 26–29 lamellae); single median row of subcaudal keeled (vs. smooth); enlarged median subcaudal scales row absent (vs. present); ventrolateral caudal tubercles anteriorly present (vs. absent); two postcloacal tubercles in males (vs. one tubercle); and yellow coloration in the subcaudal region absent (vs. present).

*Cnemaspissimilan* sp. nov. is distinguished from *C.huaseesom*Grismer et al. 2010 by having maximum SVL 48.1 mm (vs. 43.5 mm); one pore-bearing precloacal scale (vs. 5–8 scales); ventral and subcaudal scales keeled (vs. smooth); single median row of subcaudal keeled (vs. smooth); ventrolateral caudal tubercles anteriorly present (vs. absent); subtibial scales keeled (vs. smooth); yellow coloration in the subcaudal region absent (vs. present); and yellow coloration in the subcaudal region absent (vs. present).

*Cnemaspissimilan* sp. nov. is distinguished from *C.kamolnorranathi*Grismer et al. 2010 by having maximum SVL 48.1 mm (vs. 37.8 mm); one pore-bearing precloacal scale (vs. six or seven scales); pore-bearing precloacal scale row absent (vs. continuous); pore-bearing precloacal scale rounded (vs. elongated); ventrolateral caudal tubercles anteriorly present (vs. absent); and ventral pattern sexually dimorphic present (vs. absent).

*Cnemaspissimilan* sp. nov. is distinguished from *C.lineatubercularis*Ampai et al. 2020 by having maximum SVL 48.1 mm (vs. 41.8 mm); seven or eight infralabial scales (vs. nine scales); one pore-bearing precloacal scale (vs. 4–7 scales); 24 or 25 paravertebral tubercles (vs. 19–21 tubercles); paravertebral tubercles randomly arranged (vs. linearly arranged); 23 or 24 lamellae under 4^th^ toe (vs. 27–29 lamellae); caudal tubercles restricted to a single paravertebral row on each side absent (vs. present); two postcloacal tubercles in males (vs. one tubercle); and yellow coloration in the subcaudal region absent (vs. present).

*Cnemaspissimilan* sp. nov. is distinguished from *C.omari*Grismer et al. 2014 by having maximum SVL 48.1 mm (vs. 41.3 mm); one pore-bearing precloacal scale (vs. 3–6 scales); 23 or 24 lamellae under 4^th^ toe (vs. 25–28 lamellae); single median row of subcaudal keeled (vs. smooth); ventrolateral caudal tubercles anteriorly present (vs. absent); two postcloacal tubercles in males (vs. one tubercle); and yellow coloration in the subcaudal region absent (vs. present).

*Cnemaspissimilan* sp. nov. is distinguished from *C.phangngaensis*Wood et al. 2017 by having maximum SVL 48.1 mm (vs. 42.0 mm); eight or nine supralabial scales (vs. 10 scales); seven or eight infralabial scales (vs. 10 scales); one pore-bearing precloacal scale (vs. four scales); 24 or 25 paravertebral tubercles (vs. 22 tubercles); paravertebral tubercles randomly arranged (vs. linearly arranged); tubercles on lower flanks present (vs. absent); 23 or 24 lamellae under 4^th^ toe (vs. 29 lamellae); caudal tubercles restricted to a single paravertebral row on each side absent (vs. present); and yellow coloration in the subcaudal region absent (vs. present).

*Cnemaspissimilan* sp. nov. is distinguished from *C.punctatonuchalis*Grismer et al. 2010 by having maximum SVL of 48.1 mm (vs. 49.6 mm); one pore-bearing precloacal scale (vs. absent); 23 or 24 lamellae under 4^th^ toe (vs. 29–31 lamellae); ventral and subcaudal scales keeled (vs. smooth); single median row of subcaudal keeled (vs. smooth); and enlarged median subcaudal scales row absent (vs. present).

*Cnemaspissimilan* sp. nov. is distinguished from *C.samui* sp. nov. by having maximum SVL 48.1 mm (vs. 42.3 mm); one pore-bearing precloacal scale (vs. 5–8 scales); enlarged median subcaudal scales row absent (vs. present); and yellow coloration in the subcaudal region absent (vs. present).

*Cnemaspissimilan* sp. nov. is distinguished from *C.selenolagus*Grismer et al. 2020 by having maximum SVL 48.1 mm (vs. 36.2 mm); eight or nine supralabial scales (vs. 10 or 11 scales); seven or eight infralabial scales (vs. 10 scales); one pore-bearing precloacal scale (vs. six or seven scales); pore-bearing precloacal scale shape rounded (vs. elongated); 24 or 25 paravertebral tubercles (vs. 16–18 tubercles); tubercles on lower flanks present (vs. absent); ventral and subcaudal scales keeled (vs. smooth); ventrolateral caudal tubercles anteriorly present (vs. absent); and subtibial scales keeled (vs. smooth).

*Cnemaspissimilan* sp. nov. is distinguished from *C.siamensis* (Smith, 1925) by having maximum SVL 48.1 mm (vs. 39.7 mm); one pore-bearing precloacal scale (vs. absent); single median row of subcaudal keeled (vs. smooth); enlarged median subcaudal scales row absent (vs. present); and ventrolateral caudal tubercles anteriorly present (vs. absent).

*Cnemaspissimilan* sp. nov. is distinguished from *C.thachanaensis*Wood et al. 2017 by having maximum SVL 48.1 mm (vs. 39.0 mm); eight or nine supralabial scales (vs. 10 or 11 scales); seven or eight infralabial scales (vs. 9–11 scales); pore-bearing precloacal scale present (vs. absent); 24 or 25 paravertebral tubercles (vs. 15–19 tubercles); paravertebral tubercles randomly arranged (vs. linearly arranged); caudal tubercles restricted to a single paravertebral row on each side absent (vs. present); and two postcloacal tubercles in males (vs. absent).

*Cnemaspissimilan* sp. nov. is distinguished from *C.vandeventeri*Grismer et al. 2010 by having maximum SVL 48.1 mm (vs. 44.7 mm); one pore-bearing precloacal scale (vs. four scales); enlarged median subcaudal scales row absent (vs. present); and ventrolateral caudal tubercles anteriorly present (vs. absent).

## ﻿Discussion

Historically, most Thai *Cnemaspis* were known from areas of limestone karsts and granitic rock formations on the mainland in western, eastern and southern Thailand (Smith 1925; Taylor 1963; Bauer and Das 1998; Grismer et al. 2010, 2020; Wood et al. 2017; Ampai et al. 2020). Only five species of Thai *Cnemaspis* have been found on offshore islands, including *C.tarutaoensis*Ampai et al. 2019 in the *kumpoli* group and four species in the *siamensis* group, *C.adangrawi*Ampai et al. 2019, *C.chanardi*Grismer et al. 2010, *C.siamensis* (Smith, 1925) and *C.vandeventeri*Grismer et al. 2010. The discoveries and descriptions of *C.samui* sp. nov. and *C.similan* sp. nov. increase the total number of Southeast Asian *Cnemaspis* to 66 species, of which 21 occur in Thailand. This also increases the number of insular species in Thailand from five to seven. Remarkably, the geographic distribution of *C.chanardi* is relatively large and discontinuous across limestone karsts and granitic formations in southern Thailand (Grismer et al. 2014). This study suggests that *C.chanardi* might actually represent a complex of species in southern Thailand. Additional data on all *C.chanardi* populations are needed to better delineate species boundaries and estimate their phylogenetic relationships within the *siamensis* group (Wood et al. in prep).

This study revealed two unrecognized species of *Cnemaspis* in granitic areas of southern Thailand, suggesting that additional sampling might reveal more species in this region. Additionally, the phylogenetic analyses of the *siamensis* group confirmed that *C.chanardi* and *C.kamolnorranathi* are strongly supported members of the *siamensis* group. Previously, Grismer et al. (2010) described *C.chanardi* and *C.kamolnorranathi* based only on a combination of morphometric and meristic characters. The phylogenetic placements shown here based on the mtDNA (ND2) of *C.chanardi* and *C.kamolnorranathi* verified the hypotheses of Grismer et al. (2010, 2014) based on morphological and color pattern characters. The phylogenetic position of *C.chanardi* is the sister species to a clade composed of *C.phangngaensis* and the new species *C.similan* sp. nov., while *C.kamolnorranathi* is the sister species to the other new species, *C.samui* sp. nov. The north-south division of the *siamensis* group shown here is concordant with previous studies (Grismer et al. 2014, 2020; Wood et al. 2017; Ampai et al. 2019, 2020; Lee et al. 2019) that revealed a northern clade of six species (*C.huaseesom*, *C.punctatonuchalis*, *C.selenolagus*, *C.siamensis*, *C.thachanaensis*,and *C.vandeventeri*) and a southern clade of nine species (*C.adangrawi*, *C.chanardi*, *C.kamolnorranathi*, *C.lineatubercularis*, *C.omari*, *C.phangngaensis*, *C.roticanai*, *C.samui* sp. nov., and *C.similan* sp. nov.). The diversification of the *siamensis* group could be linked to the timing of sea level fluctuations that exposed the dispersal corridors between mainland and offshore islands of the Sunda Shelf (Voris, 2000; Sathiamurthy and Voris 2006; Woodruff, 2010). Additional field surveys in unexplored and overlooked areas, particularly in both limestone karst and granitic formations, are needed to better evaluate species diversity and further understand the complex biogeography of *Cnemaspis* in Thailand and adjacent areas.

## Supplementary Material

XML Treatment for Cnemaspis samui

XML Treatment for Cnemaspis similan

## ﻿Appendix I. List of comparative specimens examined

*Cnemaspisadangrawi*: Thailand, Satun Province, Mueang Satun District, Ko Adang: ZMKU R 00767 (male holotype), ZMKU R 00769–70, THNHM 28206–09 (6 males), ZMKU R 00768, ZMKU R 00771 (2 females); Thailand, Satun Province, Mueang Satun District, Ko Rawi: ZMKU R 00773, ZMKU R 00775, THNHM 28210 (3 adult males), ZMKU R 00774, THNHM 28211 (2 females).

*Cnemaspischanardi*: Thailand, Trang Province, Nayong District, Ban Chong: THNHM 06983 (male holotype); Krabi Province, Klong Thom District: THNHM 012439–40 (males); Mueang Krabi District: THNHM 012436–37 (males), THNHM 012438 (female); Nakhon Si Thammarat Province, Tha Sala District: THNHM 020992 (male); Lansaka district: THNHM 014111 (immature male); Noppitam district: THNHM 013838 (male), THNHM 010705 (male); Surat Thani Province, Mu Ko Ang Thong, Mueang Surat Thani District: THNHM 016074 (female).

*Cnemaspishuaseesom*: Thailand, Kanchanaburi Province, Sai Yok District, Sai Yok National Park: THNHM 15909 (male holotype).

*Cnemaspislineatubercularis*: Thailand, Nakhon Si Thammarat Province, Lan Saka District, Wang Mai Pak Waterfall: ZMKU R 00828 (male holotype); ZMKU R 00821–31 (males); THNHM 28694–95 (males); ZMKU R 00826 (female); THNHM 28696–97 (females); ZMKU R 00832–35 (females).

*Cnemaspisniyomwanae*: Thailand, Trang Province, Palean District, Thum Khao Ting: THNHM 15909 (female holotype).

*Cnemaspispunctatonuchalis*: Thailand, Prachuap Khiri Khan Province, Thap Sakae District, Huay Yang National Park: THNHM 02001 (male holotype).

*Cnemaspissiamensis*: Thailand, Nakhon Si Thammarat Province, Lan saka District: THNHM 013828 (male); Tha Sala District: THNHM 018265 (male); Chumpon Province, Mueang Chumpon District: THNHM 0372 (male); Phato District: THNHM 01086 (male); Surat Thani Province, Vibhawadee District: THNHM 01084 (female); Mu Ko Ang Thong, Mueang Surat Thani District: THNHM 015624 (female).

*Cnemaspisvandeventeri*: Thailand, Ranong Province, Kapur District, Klong Naka: THNHM 08261 (male holotype), THNHM 08260 (female).

## Supplementary materials

Supplementary material 1 Table S1 Data type excel file Explanation note Significant *p*-values of the ANOVA analyses (Tukey’s HSD; *p*<0.05) from 15 size-corrected morphometric measurements of nine OTUs of *Cnemaspissiamensis* species group. HD, ED and EE were not significantly different from one another. Measurement abbreviations are defined in the material and methods. https://binary.pensoft.net/file/760595This dataset is made available under the Open Database License (http://opendatacommons.org/licenses/odbl/1.0/). The Open Database License (ODbL) is a license agreement intended to allow users to freely share, modify, and use this Dataset while maintaining this same freedom for others, provided that the original source and author(s) are credited.Natee Ampai, Attapol Rujirawan, Siriporn Yodthong, Korkhwan Termprayoon, Bryan L. Stuart, Perry L. Wood Jr, Anchalee Aowphol
